# Supplementary material for: Undervalued essential work and lacking health literacy as determinants of COVID-19 infection risks: a qualitative interview study among foreign-born workers in Sweden
Source: BMJ Open. 2023 Dec 12;13(12):e069838. doi: 10.1136/bmjopen-2022-069838 (PMC10729212; doi:10.1136/bmjopen-2022-069838)
Supplement: Supplementary data [file bmjopen-2022-069838supp001.pdf]

Appendix I

Descriptives of the study participants

**Occupations of the workers**
Tram or bus drivers (n=3)
Taxi drivers (n=3)
Property caretakers (n=2)
Cleaners (n=3)
Pizza bakers (n=4)
**Union representatives and their main occupation**
Transport union: Bus drivers (n=1)
Transport union: Taxi drivers (n=1)
Property service union: Property caretaker (n=1)
Property service union: General cleaners (n=3)
**Country of origin – workers**
Turkey (n=2)
Syria (n=4)
Iraq (n=2)
Palestine (n=2)
Somalia (n=5)
**Country of origin – Union representatives**
Sweden (n=3)
Norway (n=1)
Iran (n=1)
Serbia (n=1)
