# Supplementary material for: Undervalued essential work and lacking health literacy as determinants of COVID-19 infection risks: a qualitative interview study among foreign-born workers in Sweden
Source: BMJ Open. 2023 Dec 12;13(12):e069838. doi: 10.1136/bmjopen-2022-069838 (PMC10729212; doi:10.1136/bmjopen-2022-069838)
Supplement: Author's manuscript [file bmjopen-2022-069838.draft_revisions.pdf]

BMJ Open is committed to open peer review. As part of this commitment we make the peer review history of every article we publish publicly available.

When an article is published we post the peer reviewers' comments and the authors' responses online. We also post the versions of the paper that were used during peer review. These are the versions that the peer review comments apply to.

The versions of the paper that follow are the versions that were submitted during the peer review process. They are not the versions of record or the final published versions. They should not be cited or distributed as the published version of this manuscript.

BMJ Open is an open access journal and the full, final, typeset and author-corrected version of record of the manuscript is available on our site with no access controls, subscription charges or pay-per-view fees (http://bmjopen.bmj.com).

If you have any questions on BMJ Open's open peer review process please email info.bmjopen@bmj.com

BMJ Open

Virus exposure risks, undervalued essential work, and health literacy – an interview study among foreign-born persons in high-risk jobs for COVID-19

Journal:	*BMJ Open*
Manuscript ID	bmjopen-2022-069838
Article Type:	Original research
Date Submitted by the Author:	04-Nov-2022
Complete List of Authors:	Söderberg, Mia; University of Gothenburg, Occupational and Environmental Medicine<br>Magnusson, Maria; Angered Hospital<br>Swaid, Juhaina; University of Gothenburg, School of Public Health and Community Medicine<br>Jakobsson, Kristina; Goteborgs Universitet<br>Rosengren, Annika ; University of Gothenburg, Department of Molecular and Clinical Medicine
Keywords:	COVID-19, OCCUPATIONAL & INDUSTRIAL MEDICINE, EPIDEMIOLOGY

SCHOLARONE™
Manuscripts

I, the Submitting Author has the right to grant and does grant on behalf of all authors of the Work (as defined in the below author licence), an exclusive licence and/or a non-exclusive licence for contributions from authors who are: i) UK Crown employees; ii) where BMJ has agreed a CC-BY licence shall apply, and/or iii) in accordance with the terms applicable for US Federal Government officers or employees acting as part of their official duties; on a worldwide, perpetual, irrevocable, royalty-free basis to BMJ Publishing Group Ltd ("BMJ") its licensees and where the relevant Journal is co-owned by BMJ to the co-owners of the Journal, to publish the Work in this journal and any other BMJ products and to exploit all rights, as set out in our licence.

The Submitting Author accepts and understands that any supply made under these terms is made by BMJ to the Submitting Author unless you are acting as an employee on behalf of your employer or a postgraduate student of an affiliated institution which is paying any applicable article publishing charge ("APC") for Open Access articles. Where the Submitting Author wishes to make the Work available on an Open Access basis (and intends to pay the relevant APC), the terms of reuse of such Open Access shall be governed by a Creative Commons licence – details of these licences and which Creative Commons licence will apply to this Work are set out in our licence referred to above.

Other than as permitted in any relevant BMJ Author's Self Archiving Policies, I confirm this Work has not been accepted for publication elsewhere, is not being considered for publication elsewhere and does not duplicate material already published. I confirm all authors consent to publication of this Work and authorise the granting of this licence.

For peer review only

Virus exposure risks, undervalued essential work, and health literacy – an interview study among foreign-born persons in high-risk jobs for COVID-19

Mia Söderberg[1], Maria Magnusson[2], Juhaina Swaid[1], Kristina Jakobsson[1,3], Annika Rosengren[4]

[1]Occupational and Environmental Medicine, School of Public Health and Community Medicine, Sahlgrenska Academy, University of Gothenburg, Sweden
[2]Angered Hospital, Hospitals West, Region Västra Götaland, Sweden
[3]Occupational and Environmental Medicine, Sahlgrenska University Hospital, Region Västra Götaland
[4]Department of Molecular and Clinical Medicine, Sahlgrenska Academy, University of Gothenburg, Sweden

Corresponding author: Mia Söderberg, Occupational and Environmental Medicine, Box 414, 40530 Gothenburg, Sweden.
Email: mia.soderberg@amm.gu.se

For peer review only

For peer review only - http://bmjopen.bmj.com/site/about/guidelines.xhtml

Abstract
Objectives: To investigate aspects of work, health literacy and living conditions as determinants of COVID-19 infection risks among foreign-born workers in non-health care occupations.
Design: Data were collected according to a qualitative design, using semi-structured interviews. Verbatim transcripts of these interviews were analysed according to systematic text condensation.
Participants: We recruited foreign-born persons working as taxi, bus and tram drivers, pizza bakers, cleaners and property caretakers, both workers (n=15) and union representatives (n=6).
Results: Three overarching themes were found: "Virus exposure at work", "Aspects of low status and undervalued work" and "Foreign-born persons' position". Sources of virus exposure seemed to mainly consist of social interactions, as most respondents were in contact with many people. Some interactions were part of the job, but several were not. The respondents also felt that despite carrying out important societal functions, their work was not valued, and their lives were not protected since working in society deemed low status jobs. There were reports of exploitation as some workers depended on their employment for a Swedish residency permit or to financially support their relatives. This dependency combined with low influence made it difficult to improve working conditions. Several respondents, furthermore, stated that they lacked access to relevant information from employers and public health organisations, since a lot of information was only available in Swedish or was disseminated through unknown channels.
Conclusions: Structural factors and lack of adapted information manifested itself as less possibilities to protect oneself against COVID-19 virus exposure. In an increasingly globalized world new widespread disease are likely to occur and knowledge is needed how to protect all workers equally. Our results are transferable to similar contexts and bring forth aspects which can be tried in quantitative studies or public health interventions.

Strengths and limitations of this study

- Recruitment of participants from sociodemographic groups that commonly decline participation in scientific studies, and the inclusion of both workers and union representatives.
- The use of qualitative methodology which increase the possibilities to explore new themes
- That the interviews were mostly conducted in the participants' native language. However, four interviews were conducted in a language (Swedish) that was neither the respondent's nor the interviewer's native language, which likely restricted a deeper understanding.
- Those who volunteered were likely a selection of more well-established persons and almost all the participants were men

Key word: occupational risks, COVID-19, health literacy, immigrant workers

Word count: 8039

For peer review only - http://bmjopen.bmj.com/site/about/guidelines.xhtml

Background

Throughout the COVID-19 pandemic, social disparities have been revealed, as markers of a low socioeconomic position, or belonging to an ethnic minority has consistently been associated with an increased risk of infection, severe illness and death from COVID-19 (1, 2). Severe disease and death in the lower socioeconomic strata depend on a higher prevalence of underlying diseases (3) but being infected is determined by virus exposure. COVID-19 is primarily transmitted through drops in sneezing, coughing or speech, or aerosol transmission, with the latter being exuberated in crowded and poorly ventilated spaces. As such, close and frequent contacts with other people is crucial for the spread of infection. In the working age population, work is a dominant source of social contacts, but with marked differences in the possibilities for the individual to protect oneself, as low-wage workers generally have less possibilities for social distancing at work or working from home (4, 5).

When COVID-19 was declared a pandemic by the World Health Organisation (11th of March 2020) some occupations, primarily in the healthcare sector, were promptly identified as high-risk occupations. Still, there are many other jobs with frequent and close physical interactions, in which virus exposure and protection possibilities have been less investigated. One review, including studies based on the early stages of COVID-19, showed an increased risk of contracting COVID-19 in abattoir workers and related food workers, as well as retail staff (6). Two Swedish register studies found the highest incidence of a positive PCR test for COVID-19 (until May and June 2020), outside healthcare jobs, among taxi drivers, pizza bakers, and bus and tram drivers (7, 8). Other occupations identified were property caretakers and cleaners. A smaller study, recording COVID-19 cases during the first 40 days since a first detected case in six Asian countries, noted the highest infection rates in taxi and van drivers and, in descending order, shop salespersons, religious professionals and construction laborers (9).

As time progressed, similar and new at-risk occupations were observed. In one register study with access to national health registers until December 2020, the highest COVID-19 mortality rates were found in taxi drivers (Hazard ratios (HR) 2.01, 95% confidence intervals (CI) 1.67–2.43, in men), followed by support staff, bus and coach drivers, sanitary workers and van drivers, adjusted for age, multiple sociodemographic factors and prior diagnoses (10). Another national register study noted the highest odds ratios (OR) for COVID-19 (positive PCR test or an ICD-10 diagnostic code U07.1) during winter–spring 2020 in bus and tram drivers, taxi drivers, food counter attendants, bartenders, shops assistants and cleaners (11). Corresponding risk occupations during the second pandemic wave (winter 2021) were public transport conductors, bartenders, travel stewards, waiters, food counter attendants, bus and tram drivers, taxi drivers and hairdressers. A report from the Swedish Social Insurance Agency, using sick leave data until March 2021, illustrated a 42% increased relative risk of long-term sick leave (>21 days) in taxi drivers, compared to all other employed persons (12). Bus and tram drivers also displayed over-risks of both short-term sick leave (42% increased risk of at least one day of sick leave) and long-term sick leave (39% increased risk). Thus, some occupations with similar characteristics consistently show an increased risk of COVID-19.

Furthermore, high infection risks seem to interplay with having an immigrant background. In Sweden, the majority of the occupations with a high occurrence of COVID-19 also employ the highest proportion of foreign-born persons, for example, pizza bakers (79%), cleaners (57%), bus and tram drivers (48%) and taxi drivers (47%) (13). A Norwegian register study evaluating occupational risks for COVID-19 in immigrants from Somalia, Pakistan, Iraq,

For peer review only - http://bmjopen.bmj.com/site/about/guidelines.xhtml

Afghanistan and Turkey found that foreign-born taxi or bus drivers, restaurant assistants and cleaners displayed a substantially higher risk of receiving a COVID-19 diagnosis than the general population. However, foreign-born persons in these occupations did not display a higher risk than other immigrants from the same country (14). A British registry study found higher mortality in COVID-19 among taxi drivers, bus drivers and cleaners, but the risk estimates decreased markedly (taxi drivers and cleaners) or were nullified (bus drivers) when adjusting for age, ethnicity, housing, living in a socio-economically vulnerable area and existing diseases (15). In Sweden, several markers of low socioeconomic status but also an origin from outside Sweden have been associated with increased mortality in COVID-19. Near doubled HRs were found in persons from Estonia, Lebanon, Syria and Turkey, with a more than three-fold mortality in immigrants from Somalia (HR 3.63, 95% CI 2.14–6.18), adjusted for socioeconomic factors and chronic diseases (16).

In addition to occupational exposures, another vulnerability may relate to access to important information or living conditions. Foreign-born workers from ethnic minorities tend to make less demands at work or to a lesser extent try to improve their working conditions (17), partly for fear of repercussions, but also because of language barriers or lack of social networks, which leads to reduced access to information on safety and rights. Such processes relate to "health literacy", a concept that can be briefly explained as the use of information to protect and improve health (18). At a basic level, it involves having adequate language skills, while the more complex features reflect an ability to acquire and apply information. Health literacy also includes an organisation’s ability to adapt appropriate support for health-promoting behaviour.

There are likely also additional risks from a higher occurrence of poor housing conditions, more commonly found among foreign-born persons. Sweden, for example, displays some of the largest within-country gaps in inequalities in housing deprivation in Europe, as around 30% of all foreign-born persons live in overcrowded households, compared to 9% of Swedish-born persons (19). If people in overcrowded households also tend to work in high-risk occupations, this implies an added risk of a circular infection pattern between the work and home environment.

Despite the higher risk of COVID-19 in several sociodemographic groups and occupations, little is known about infection risks in day-to-day situations, as perceived by the workers themselves. An American interview study of fast-food workers (n=15) (20) illustrated aspects of occupational risk, such as customers refusing to wear facemasks or subjecting workers to verbal abuse or threats when asked to practice social distancing or to wear a facemask. Another interview study including bus drivers, delivery workers and police staff noted late or lack of access to protective equipment and difficulties in distancing themselves from other people (21). Otherwise, there is little self-reported information on occupational infection risks in occupations outside the healthcare sector. The aim of this study is to increase the understanding of pathways for COVID-19 infection in foreign-born persons in non-health care occupations, in both their work situation and in their overall life situation.

Methods

Recruitment of participants

Based on previous reports of Swedish occupations with a high prevalence of COVID-19, we chose to investigate the study objective among persons in the following occupations: pizza bakers, taxi drivers, bus and tram drivers, cleaners and property caretakers. Initially we only

For peer review only - http://bmjopen.bmj.com/site/about/guidelines.xhtml

included workers, but as the project progressed, we added union representatives who might contribute with an overview, while also having experienced the working conditions themselves. The participants were recruited through social networks, referred to as convenience sampling, through 1) colleagues at the Department of Occupational and Environmental Medicine, Sahlgrenska University Hospital, and 2) health guides at the East Gothenburg Health centre; Juhaina Swaid (co-author) and Saido Mohamed. Health guides in Sweden have a broad range of tasks such as facilitating communication between organisations and citizens, disseminating knowledge about how people can promote their health, and navigating the healthcare system in order to ensure that everyone in society has access to the same information. The union representatives 3) were recruited by emailing each union's main contact persons. When contact was established, the person in question sent an invitation email to either a selection of potentially interested union workers or a global email list.

Patient and public involvement

No patient involved.

Data collection

The interviews with the workers were conducted from March 2021–March 2022, while all the union representatives were interviewed during February–March 2022. The interviews were conducted according to both semi-structured methodology with a set of both open-ended questions to encourage the participants to freely describe their situation, and questions about specific work environment aspects, for example, the number of social contacts the participants had during an average working day. The interview guide comprised three main topics: 1) work-related virus exposure and protective measures and 2) access to information and 3) living conditions. Sample of interview questions, (Infection risks and preventive measures): "How often do social contacts at work require other people to stand very close to you and shout in order to overcome the surrounding noise?", "What methods have you used to protect yourself from exposure to infection?", (access to information): "How do you obtain information about COVID-19?", (living conditions): "Are there any reasons why you would not call in sick if you had COVID-19 symptoms?". We also included the question "What do you think is the main reason for the spread of COVID-19?".

The interviews with the union representatives included the same questions as for the workers but the questions were asked from the perspective of both their own working environment and how they perceived the work conditions of the members they represented. After the 15th interview with the workers, the content became repetitive, and the data was perceived as saturated, and the recruitment ended. All union representatives who reported an interest in participating were interviewed. Expect for Juhaina Swaid (JS), none of the authors had any prior social contact to any of the participants. JS had encountered some of the workers prior to this study through her work as a health guide.

Language considerations and transcription

The interviews were carried out by JS (native language Arabic), Saido Mohamed (SM) (native language Somali) and Mia Söderberg (MS) (native language Swedish). The interviews by each interviewer and language are shown in Figure 1. Fifteen workers and six union representatives participated. The pizza bakers were all self-employed or worked in small family-owned business, which is common in this occupation in Sweden, and we were unable to find any union representatives for this occupation. Most workers were men (n=12), while the union representatives comprised three male and three female participants. The

For peer review only - http://bmjopen.bmj.com/site/about/guidelines.xhtml

union representatives worked with union assignments one to four days per week; the remaining time they spent working in their main occupation. The unionised members they represented ranged from 300 to 7000 members. Further descriptive information about the study participants can be found in Appendix I.

Figure 1. Languages used during interviews

None of the 15 workers had Swedish as their native language, out of these five interviews were conducted in Arabic by JS and four interviews in Somali by SM, i.e., an interview situation in the native language of both persons. The remaining six workers were interviewed in Swedish, of which four were conducted by JS, i.e., in a language which was neither the interviewer's nor the respondent's native language. With this in mind, JS revised the transcript of these interviews in order to clarify aspects that may not be apparent from the transcripts. The six union representatives were interviewed in Swedish by MS. Swedish was not the native language of three union representatives, but their language level was perceived as equal to a native Swede (native language: Norwegian), or as an advanced Swedish speaker (native languages: Serbian and Persian). The interviews in Arabic and Somali were translated into Swedish by professional translators and all 21 interviews were then transcribed verbatim by the same company that was contracted to translate the interviews. The translated transcripts were revised by JS (Arabic interviews) and SM (Somali interviews) to evaluate whether they corresponded with their perception of what emerged during the interviews.

Analyses

The analysis of the data was mainly conducted by first author MS and second author Maria Magnusson (MM), who are both experienced in conducting qualitative studies. MM has particular experience with systematic text condensation as proposed by Malterud (22), which was the chosen analysis method of this study. This methodology is characterised by paying particular attention to the participants' experiences and perspectives, while emphasising an understanding of the underlying processes. Accordingly, MS and MM started by reading the entire transcript of all the interviews, without singling out meaningful information, in order to get the overall gist of the material. Both authors then re-read the printed transcripts and marked all content that was perceived as meaning bearing units with focus to the study objectives. Other content was marked to be re-evaluated later.

In the next step the meaning units were revised and labelled into broad categories as either relating to work, general living conditions, aspects of information or being foreign-born. From this step onwards, JS participated in the process with interpretations and her professional and cultural knowledge. The results were then presented and discussed among all the authors and condensed into main themes with underlying sub-themes. The senior researchers, Kristina Jakobsson (JK) and Annika Rosengren (AR), contributed with their knowledge of research in public health and occupational medicine. As a result of the discussion among all authors, access to information was changed from being a main theme to a sub-category as an aspect of being foreign-born. The sub-themes were also evaluated with consideration to how often a certain theme was mentioned, for example if the respondents indicated if it was something only they themselves encountered or if it seemed common among the unionized members the union representatives represented.

For peer review only - http://bmjopen.bmj.com/site/about/guidelines.xhtml

Results

Our analyses resulted in three overarching themes: 1. Virus exposure at work, 2. Aspects of low status and undervalued work and 3. Foreign-born persons' position, with the main and sub-themes as presented in Table 1.

Table 1. Main and sub-themes

Main Themes	Sub-themes	
1. Virus exposure at work	1.1 Hard-to-avoid contamination risks	Social contacts<br>Surfaces
	1.2 Lack of safety culture	Employees<br>Employers<br>Employer liability
	1.3 Contributing to the spread of disease	Social contacts over many venues<br>Contact with vulnerable persons
2. Aspects of low status and undervalued work	2.1 Fulfilling an unappreciated societal role	Sanitation<br>Public transport<br>Food services
	2.2 Occupational status influences workers' value and safety	Interventions deemed too expensive<br>Unequal protection
	2.3 Low authority over their own work	Lack of influence<br>Distance from decision makers
3. Foreign-born persons' position	3.1 Health literacy	Workers<br>Employers<br>Organisations
	3.2 Exploitation	Need the job to financially support others<br>Need the job for Swedish residency<br>Unawareness of regulations or rights
	3.3 Protective directives were not adapted for persons outside the majority	Overcrowded living conditions<br>Different social habits<br>Industries in which directives do not apply

1. Virus exposure at work

Due to the inability to work from home, the nature of the work tasks and the organisational environment, the participants felt it was near impossible to avoid exposure situations. Sub-themes for "Virus exposure at work" were sorted into "Hard-to-avoid contamination risks", "Lack of safety culture" and "Contributing to the spread of disease".

1.1 Hard-to-avoid infection risks

Most of the participants encountered many close contacts over a workday with people for whom service was part of the job, with taxi, tram and bus drivers reporting several aspects of close physical interactions. While most taxis and buses had a protective screen installed, the drivers sometimes conducted mobility services for citizens with reduced functional ability, for example, the elderly, physically disabled persons, or school children with special needs. These passengers often needed support getting in and out of the vehicle, as well as with fastening their seatbelts. One taxi driver estimated that around half of her mobility service assignments involved closeness to the point of touching the passenger.

For peer review only - http://bmjopen.bmj.com/site/about/guidelines.xhtml

Taxi driver: *"(Interviewer): Are there people with disabilities or elderly people...?) (Taxi driver): There are some who are ill. I help elderly people who can't sit down, or put on their seat belt or walk up the stairs: (Interviewer): Do you support them? (Taxi driver): Maybe I'll hold their hand, yes."*

Bus and tram drivers reported being protected during periods when the front doors were disabled and protective screens were installed in the driver's area. The bus union representative, nevertheless, remarked that some companies did not re-introduce the protective screens during the second wave (winter 2021) and demanded that the passengers entered through the front doors in order to facilitate ticket inspection by the driver. This meant that many people came into contact with the driver. One bus driver estimated that when the restrictions were relaxed, she interacted with approximately 250 passengers during an average working day.
Union representative for bus drivers: "*For me, the passenger is the cause of infection. I drive you to work, to your exercise, to the store, to the health centre. One guy was standing and talking to me last week. He was going to the health centre for a COVID test! What the h*ll are you standing here saying, I thought*" (comment: interview conducted in February 2021).

The pizza bakers, who were self-employed or worked in a small business, would make rules for customers to wait outside for take-away food or install protective screens near the counter. The property caretakers rarely met people when tending to residential yards or stairwell areas during their working day, and if they needed to enter an apartment, they would make an appointment when the occupants were away or request that they stayed in another room.

Many respondents also reported social interactions which were not directly part of their work tasks. The cleaners worked in crowded venues such as schools, hospitals and shopping centres. Of particular note were the hospital cleaners who came into contact with patients who wanted to engage in small talk or needed help, or medical staff who refused to leave their offices.
Hospital cleaner: *"The patients need help. We say no! This is not part of our job. But if they are old and can't find the buttons* (comment: the buttons used to call for help or operate their bed), *then we will show them the buttons. Some of the patients are old, then you have to stand close and speak loudly."*
Hospital cleaner: *"Always when I enter this office, the doctor is sitting there. He doesn't want to leave. So I wipe a bit there* (comment: the participant cleans the area around where the doctor is sitting).
When communicating with colleagues, most participants could keep their distance or turn their faces away if they were too close. As an exception, the hospital cleaners worked together in cramped spaces and sometimes removed their facemasks in noisy environments or when talking to a co-worker with limited Swedish language skills in order to improve communication with facial expressions.

Additionally, according to the union representatives for cleaners, both they themselves and the workers they represented frequently travelled by public transport between assignments as they did not own a car, or to avoid parking fees and traffic jams. This made them feel exposed to additional risks since facemasks were not mandatory on public transport in Sweden. The bus and tram drivers would also use public transport to travel to the start and end stations of their shifts.

For peer review only - http://bmjopen.bmj.com/site/about/guidelines.xhtml

Union representative for cleaners: *I myself have a car but I don't want to drive. You end up in traffic jams and you can't find a parking space. There's a lot of time pressure in our work… Therefore, people are forced to go for example by train, by bus… I can see that the people who take public transport do not use facemasks."*

Many of the participants, particularly, cleaners and property caretakers, handled tools or surfaces that had been touched by many other people. The hospital cleaners not only came into frequent contact with surfaces touched by patients and hospital staff in wards, waiting rooms and toilets, they rarely had time to sanitise their cleaning equipment more than once a week. Property caretakers operated over large areas and handled waste. Taxi, bus and tram drivers would clean the driver's area or vehicles as often as possible, with professional cleaning carried out every night (buses) or once a week (taxis). All pizza bakers reported that they frequently sanitised their work areas and surfaces touched by customers.

1.2 Lack of safety culture

Most workers reported trying to work in a safe manner. Yet, some also stated that safety measures were strongly requested at the beginning of the pandemic, but became less important as time passed, due to stress, feeling restrained by cumbersome protective equipment or generally being less worried about COVID-19. In terms of the protective measures provided by the employers, reports varied. Some participants' employers seemed to have enforced safety procedures and provided their workers with personal protective equipment immediately, while other employers delayed providing personal protective equipment until autumn 2020, and only when they had been pressured to do so.
Bus driver: "*No, we didn't receive any recommendation during April–May 2020. No one warned us about this. It came after the numbers of infected people increased. Then the company and the municipalities began to take action and give recommendations. They started with it after the month of July* (comment: July 2020).

The cleaners also reported late interventions and that some of them did not receive facemasks until October 2020, despite working close to patients and COVID-19 wards.
Hospital cleaner: *"In the beginning they sent us to a department called ICU and it was full of patients who had had covid, we went there without protection, without mouth protection"* (comment: the unit was cleared of patients before cleaning).
Hospital cleaner: *The emergency department built a barrack for examinations. Those who came were unwell. They had, Corona or fever, or something. We asked why we are cleaning there - it's a risk. He* (comment: their manager) *says you must do it, there is no risk."*

Two union representatives recounted how some employers would object about having to purchase facemasks, since the cleaners already had plastic gloves. Additionally, according to the union representatives, many employers appeared to not have conducted any protective actions at all to avoid expenditure on a "soon-to-be-over" crisis. One representative estimated that around half of the cleaners in her geographical district, i.e., all cleaners, not just members, never received any protection at all (as of March 2022). However, she did not mention whether these cleaners worked in crowded environments or in empty spaces, for example, warehouses.

The participants speculated that safety at work related to employers lacking knowledge of best practice, not having enough resources for protective actions, or simply lacking the motivation to protect their staff. Some participants felt that employers applied practices which looked good "on paper" but neither cared or even contradicted safety protocols. For

For peer review only - http://bmjopen.bmj.com/site/about/guidelines.xhtml

example, staff meetings would conform to the regulation of a maximum of eight persons, yet lunch breaks were knowingly scheduled in such a way that the number of cleaners in a small lunchroom would far exceed eight people.
Hospital cleaner: *"They say we can't have staff meetings because of being to many* (comment: more than 8 people, the stipulated limit of people at social gatherings at the time). *When we're in the lunchroom there are more than eight people there.... but I have to sit and eat!"*
The hospital cleaners also mentioned that the managers ignored staff who came to work with obvious COVID-19 symptoms. Some cleaners, taxi drivers and bus drivers also mentioned that those who spoke up were punished with "graveyard shifts" or fewer work hours.

In mobility services carried out by taxi and bus drivers, unclear employer liability made it difficult to enforce improved safety conditions, as the services were mainly bought by the local county, while one company might own the vehicle and another company employ the driver. One union representative recalls how she early during the pandemic emailed the transport companies, the contracting unit and the borough politicians that make decisions regarding mobility services, but she did not receive any reply from any of them. There was also uncertainty regarding who was responsible for protecting cleaners working at shopping centres or schools from social interaction with pupils, the parents of pupils and shop customers.

1.3 Contributing to the spread of disease
Some work tasks and company directives made the participants feel that they themselves contributed to disease exposure to other, sometimes vulnerable, people. The general cleaners visited a large number of venues and would meet many people. The hospital cleaners served many wards, interacting with both healthcare staff and patients. During times when the rule of a maximum of three passengers was relaxed, taxi drivers conducting mobility services drove up to four passengers in their cars. The passengers, who were sometimes perceived as a risk group due to their old age or poor health, came from many different venues, for example, community centres, and then shared one car.

2) Aspects of low status and undervalued work
In addition to unavoidable disease exposure at work, there was vulnerability from the position of working in a job with low status, with the sense that their work or safety was not important and valued, despite providing important societal services. Low job status also translated into having little control over the work situation. Such aspects were sub-categorised into "Fulfilling an unappreciated societal role", "Occupational status influences workers' value and safety" and "Low authority over their own work".

2.1 Fulfilling an unappreciated societal role
Most of the participants had jobs which they felt fulfilled societal bearing tasks, and some participants had enhanced responsibilities during the pandemic. Cleaners uniformly reported increased volumes, frequency and details of cleaning tasks. Most received considerable appreciation and felt pride in their contribution to battling COVID-19 but were worried that their hard work would be forgotten once the pandemic was over. Several cleaners also expressed anger that contractors or employers wanted them to work more but without wanting to pay them for the extra work. Similarly, bus, tram and taxi drivers felt that their work was undervalued and that people in general do not realise that their work upholds the transport infrastructure, facilitating travel for the general public, other essential workers and persons with poor health.

For peer review only - http://bmjopen.bmj.com/site/about/guidelines.xhtml

Union representative for bus drivers: "*We feel very forgotten in this. If we stop all traffic, then half of Sweden stops because then the health care workers can't reach their destination, then the teachers and students can't reach their destination. We are equal, we are not health care staff, but we are important in society.*"

2.2 Occupational status influences workers' value and safety

According to several union representatives, there seems to have been a balancing act between safety and revenue. For some taxi drivers, the protective screens and restrictions regarding front seat passengers were removed around September 2020 and were not reinstated during the second wave (winter 2021), since that would require extra cars and staff, i.e., less profit. Initially, some bus companies resisted introducing facemasks so as to not "scare passengers". With time, drivers were ordered to wear a facemask or visor, but this posed new problems as they would fog-up glasses, and protective screens and visors could cause mirror reflections, endangering driving. One union worker reported that some buses received a driving ban due to these mirror reflections but were put into service anyway. The union negotiated restrictions regarding the area around the driver, but some companies resisted as it would then not be possible to carry out ticket inspections, leading to losses from lost revenue.

Union representative for bus drivers: "*They lost 1.6 billion* (comment: in Swedish krona) *last year. If you divide 1.6 billion by the number of drivers, you know the price of my life. Someone who sits in an office decides they want to gain money in this way.*" (Comment: a loss equal to around USD 160,400,000, since ticket inspections was temporary stopped).

Union representative for taxi drivers: *Yes, but it's clear that this is a huge cost because you need to have more cars available so that in November* (comment: November 2021) *it was decided that front seats should be made available. On 23 December* (comment: December 2021)*, I called my boss and said, "I won't place anyone in the front seat". He completely panicked and said, "you can't do that", "well yes I actually can because it's about my life and health."*

Another example was hospital cleaners who felt they should be offered quick antigen tests at work for free, just like healthcare staff, since they work in the same premises. Instead, during the first wave of the pandemic, they had to contact their local healthcare centre for a test and wait at home for the results (1–4 days). Since paid sick leave was not on par with their salary, this also meant a loss of income.

2.3 Low authority over their own work

Most of the workers stated that they had limited possibilities to influence their work environment or make themselves heard by management or employers. In several instances the people who made the decisions on work and safety rules seemed to be far removed from the day-to-day work environment, with little knowledge of the actual work content. Several union workers expressed anger that those in charge worked from home in a safe environment and did not listen to workers or union representatives or looked down on them and perceived them as "dumb", increasing the feeling of lost work control and low status.

Union representative for taxi drivers: *It's about us being just taxi drivers. Yes, and that's the hierarchy, yes… and it's really sad to say it, but that mentality is out there* (comment: with the decision makers)*, "they're dumb taxi drivers", it's impossible to get away from that."*

3) Foreign-born persons' position

In addition to working in high risk and low status occupations, there were themes that were directly related to an immigrant background. These themes touched issues such as inadequate

For peer review only - http://bmjopen.bmj.com/site/about/guidelines.xhtml

"health literacy" in both workers and those who are responsible for disseminating information, "exploitation" of a vulnerable position, and that "protective directives were not adapted for persons outside the majority society".

3.1 Health literacy

Several participants reported that they, their colleagues or union affiliated members had insufficient language skills to properly understand information in Swedish from employers and Swedish health officials, especially if such information was formulated in a bureaucratic style, or with ambiguous phrasings. Because of the initial lack of information in languages other than Swedish, many respondents did not use official channels, even when translated information in their native language eventually became available.

Pizza baker: "*What I don't understand is – there's no requirement for facemasks. And then they say that we should wear facemasks when it's… high time? Or when there is rush hour? I cannot know what time it is rush hour… should I wear a facemask or not?*"

According to the union representatives, as of winter 2022, most employers still seemed to only distribute information in Swedish, which they speculated was due to lack of time, insecurity over consequences if mistranslating, or simply lack of caring. There were also arbitrary methods in information outreach. Taxi, bus and tram drivers and property caretakers, whose routes and assignments need to be coordinated, already used staff apps or web platforms, which employers would use to spread COVID-19 information. The general cleaners did not have any global communication tool. Instead, they mainly communicated with managers or employers using their private mobile phones. All interviewed pizza bakers worked in small businesses, where verbal communication dominated. Otherwise, most participants described leaflets on staff noticeboards, mostly in Swedish, as a common method. The participants figured that their employers knew that some workers couldn't understand this kind of information, but that it "looked good on paper". Some of the respondents received oral information at staff meetings, but these were also mostly conducted in Swedish.

Hospital cleaner: *"In our job there are many foreigners. They don't understand the language very well. Sometimes they sit and listen* (comment: during staff meeting) *but they don't understand anything."*

Virtually none of the workers mentioned official health outlets as a source of information and, for most of them, the Public Health Agency of Sweden, the main distributor of COVID-related information in Sweden, appeared to be unknown, at least during the first phase of the pandemic. The workers primarily used social media pages by prominent people or news channels from their country of origin, or the social networks of friends and relatives. Self-employed pizza bakers would consult networks of other restaurant workers and owners. Some participants mentioned institutions which they associated with health care, which they were familiar with and trusted, such as their local healthcare centre or pharmacist.

Property caretaker: (Interviewer): *"Do you trust the authorities?"* (Property caretaker) *"No I don't. I am newly arrived in Sweden, there are many websites that are linked to the authorities which I am not aware of. I don't know who to contact. I might contact the health centre or the pharmacy."*

Bus driver*: The Swedes are a people who trust the government. The Swedish media has not explained clearly and in a good way about corona for migrants or new arrivals, the consequence of this was that a large part of them have died from corona."*

For peer review only - http://bmjopen.bmj.com/site/about/guidelines.xhtml

Despite low usage, some participants still ranked Swedish authorities as the most trustworthy source of information and most participants were aware that social media or personal networks contained a lot of misinformation. It should also be noted that some participants regarded the information they received from their employer as being adequate.

In response to an open question on how information dissemination could be improved, several suggestions were made. There was primarily a need for information in languages other than Swedish and that important information for people who run their own business could be sent by post to anyone registered as self-employed. Since some immigrants, especially older people, have difficulties reading, another suggestion was to create audio information, which could be disseminated via audio files on government websites. During times of less strict regulations, information could be disseminated at staff meetings. One union representative for cleaners, for example, organised large outdoor meetings, which he felt were very appreciated. Additionally, several participants expressed confidence in health centres or pharmacies, since they had previously had a positive experience of them, and they were located near their homes.

3.2 Exploitation

One of the most severe observations was vulnerability based on employment as a prerequisite for maintaining a Swedish residency permit, since obtaining employment is one of few methods to legally remain in Sweden if your asylum visa has been terminated or denied. Furthermore, as explained by the bus driver union representative, Sweden has implemented vocational training programmes specially designed for immigrants to help newly arrived persons get a job. Thus, there is a high proportion of foreign-born persons without permanent residency in some occupations, which the union representatives felt were taken advantage of. Union representative for bus drivers: *We often notice that newly arrived persons attend the SFI* (comment: Swedish for immigrants) *training programmes and the vocational bus training programme. You are then completely dependent on your boss to get a residency permit. They would rather drive with the wrong salary to get a residence permit. Because of this I have chosen representatives against these employers who actually choose to exploit these people. This is almost modern slavery, you do as I say, or you go home"* (comment: she has chosen specific union representatives to combat these exploitation issues).

Additionally, many employers seemed aware that some immigrants needed to work in order to financially support their relatives in Sweden or in their country of origin, which might also be taken advantage of, as speaking up against poor working conditions could result in less working hours or the worst shifts. Such needs were also mentioned as a contributing reason for not joining the union since the union would not accept unregulated overtime.

3.3 Protective directives not adapted for persons outside the majority

There was a sense that much of the safety advice disseminated by the Public Health Agency of Sweden, such as working from home or avoiding public transport, was not adapted to the participants' life situation. One new theme which arose, for which there was no directly adapted information, was large social gatherings with persons from the same country, often at privately arranged cafés or at local venues. Other examples were large gatherings in cramped local food stores that provided groceries from the participants' home region, mostly owned by other immigrants.

One aspect of interest in this study, linked to the spread of disease, was overcrowding. However, none of the interviewed persons could be defined as living in overcrowded

For peer review only - http://bmjopen.bmj.com/site/about/guidelines.xhtml

conditions. Also, only two workers and one union representative reported limited finances as an obstacle to sick leave. Otherwise, most of them reasoned that health was more important than money or that they were financially secure enough to go on sick leave.

Discussion

The results from this interview study resulted in three overarching themes. The first theme concerned virus exposure at work, through large numbers of social contacts with persons using both the services that the participants provided, but also social interactions not related to work tasks, as well as a faltering safety culture. The second theme was a sense of labouring in a society-deemed low status occupation where the participants' work or they themselves were not adequately appreciated or protected, despite carrying out important societal functions. The third theme revolved around aspects of being foreign-born where a lack of health literacy meant that many people had limited access to essential crisis and safety information. A vulnerable position in the labour market or residency in Sweden could also lead to exploitation and made it difficult, if not impossible, to speak out against hazardous working conditions.

Virus exposure at work

International studies have consistently found that working in a low-status occupation is associated with an increased risk of COVID-19 (23). In this respect, the media and public health organisations have mainly focused on the health and social care sector, but other industries and large occupational groups have also suffered increased risks (24). In this study, we took a closer look at five occupations in which a high incidence of COVID-19 has been observed in Sweden and several other countries (8, 14, 25), but with less information on the determinants of potential infection over a typical working day. Weighing in hard-to-avoid sources of potential infection and poor organisational climate, cleaners, taxi, bus and tram drivers appeared to be the most at-risk occupations, with many physically close social contacts. This may explain the high infection and mortality risk among bus, tram and taxi drivers, which seems to remain high over time throughout the pandemic (11, 12). Close contacts when taxi drivers conducted mobility services also meant increased infection risks for passengers, who frequently constituted risk groups from based on old age and poor health.

A few previous studies on taxi- and public transport drivers have presented similar findings (26), while little is known about cleaners, property caretakers and pizza bakers, the latter being best compared to fast-food workers from an international perspective. An American study, using questionnaires (n=417) and in-depth interviews (n=15) with fast-food workers (20) illustrated several occupational hazards linked to an employer's inadequate safety culture, such as lack of facemasks (40% of workers needed to buy their own facemasks), with around 10% of staff being unable to afford them. Many co-workers or customers did not wear facemasks, with frequent incidents of threats and verbal abuse from customers when trying to make them adhere to safety protocols (20). However, the fast-food workers in our study were mostly self-employed and were seemingly able to protect themselves more effectively.

Aspects of low status and undervalued work

In the midst of the pandemic, the inequities between different groups of workers became apparent, as workers in higher status jobs could shield themselves by working from home, while those in already precarious jobs needed to go to work, exposing themselves to the risk of infection. In most countries, including Sweden, healthcare workers were hailed as heroes during the pandemic. However, the occupations in our study, which also carry vital societal functions, did not receive the same widespread acclaim. Bus, tram and taxi drivers sustained

For peer review only - http://bmjopen.bmj.com/site/about/guidelines.xhtml

the transport infrastructure and cleaners had an enhanced role since increased cleaning was regarded as an important measure to prevent the spread of COVID-19. One qualitative study (21), conducted among bus drivers and transport workers, found similar themes as this study, primarily a sense of being unprotected at work, late or no introduction of protective equipment, as well as comparing themselves with other essential workers but without the same level of appreciation.

The definition of a low status job is often related to a lack of longer formal training requirements or the value of performed work tasks. Most of the investigated occupations can, from this definition, be defined as society deemed lower status jobs. Finding new staff is often also relatively easy, which may further devalue the workers' value. As such, one of our participants reported that their employer calculated the financial loss relating to protective actions and weighted this against the safety of their staff. The hierarchical distance between the workers, decisions makers and employers also resulted in the workers and union representatives feeling frustrated that the people in authority lacked knowledge of their work and did not make decisions on best practice.

Foreign-born persons' position

International studies have almost unanimously concluded that immigrants in low status jobs appear to be most at risk of contracting COVID-19. The labour market in Sweden is not only gender segregated, but an emerging public health issue is an increasing ethnic segregation, as low status occupations are more and more dominated by foreign-born workers, and that in such occupations foreign-born workers tend to be assigned the worst tasks (27). One of the most serious findings, reported by the union representatives, was exploitation, as there were some employers who abused the fact that a Swedish residency permit could be tied to employment. Similar findings of a precarious position regarding a residency permit and links to exploitation and poor working conditions have been found in many other countries (28).

Another finding was the dissemination of crisis information and lack of two-way health literacy. When COVID-19 was declared a full-scale pandemic, it dominated the news flow worldwide and awareness of the disease was high, but crucial information did not reach everyone equally. An EU-funded consortium (COVINFORM) concluded that morbidity in COVID-19 partly depended on "Vulnerability in relation to communication", meaning lack of access to, or poorly adapted, information (23). A Swedish study has illustrated that in geographical areas marked by low socioeconomic status, there is a higher use of social media, foreign news media or social networks as the main source of information, especially in individuals lacking Swedish language proficiency (29). This difficulty to access important societal information, sometimes referred to as "the knowledge gap", has been observed in several other countries, in which foreign-born persons have more difficulties accessing information based on a lack of social connections and trust in the new country (30).

Health literacy often focuses on the skills of the recipient, but it is indeed a two-way process in which the disseminator, in this context, employers and the Swedish authorities, have a responsibility for disseminating information in a manner that is both understood and adapted to the communication patterns of the receiver. This is not only the ethically correct approach but also has a bearing on fundamental human rights in an individual being given the opportunity to protect their health. In order to achieve this, those persons towards whom the information is directed must be given the opportunity to influence and be provided with communication channels to those persons responsible for compiling and disseminating

For peer review only - http://bmjopen.bmj.com/site/about/guidelines.xhtml

information. In this way, knowledge of communication channels, special needs and unknown risks can increase.

Other considerations
Pre-pandemic, foreign-born persons already displayed worse health and a shorter life expectancy than the general population, and during the pandemic also constituted the largest socio-demographic risk group for COVID-19 mortality (16). Thus far, little is known about long-term health after COVID-19, but workers in at-risk occupations have been more exposed to potential infection and may face higher risks of reduced work ability from post-COVID (31). There has therefore been a debate as to whether the long-lasting health risks of COVID-19 are severe enough that health organisations such as the WHO have a responsibility to enforce directives to protect workers (32).

Furthermore, the purpose of our study was to develop knowledge that in the long run could contribute to better interventions, such as improved and adapted support at both work and in a person's private life, and increased opportunities for people to protect their health. One example could be the improvement of adapted information, for which the participants provided several suggestions: primarily, information translated into more languages, access to information in audio format or perhaps increased dissemination from local healthcare centres or pharmacies.

Lastly, there is a risk that this kind of study may support a discourse of blame or stigmatisation. Even before the COVID-19 pandemic, the public debate concerning certain demographic groups or vulnerable geographical areas was characterised by a negative and one-sided approach. Such problematic images are at risk of being exacerbated during the pandemic, as certain urban areas or ethnic groups displayed a higher occurrence of COVID-19, at the expense of understanding the underlying causes. It is therefore important that future studies acknowledge the existing structural inequalities in order to avoid that groups with limited resources to govern their life situation, are being blamed for increasing the spread of the disease.

Limitations
Based on the language proficiency of the respondents, we only conducted interviews in Swedish, Arabic and Somali. Four interviews were conducted in a language (Swedish) that was neither the respondent's nor the interviewer's native language, which likely restricted a mutual deeper understanding. Furthermore, it is likely that those who volunteered were a selection of people more well-established in Swedish society and that we failed to recruit those people most exposed to risk. Almost all the participants were men, because most of the selected occupations are mainly held by men. Nevertheless, foreign-born women more often report poorer working conditions or leaving the labour market prematurely, compared to both foreign-born men and Swedish-born women (27). Because of the male majority, aspects of dual vulnerability based on gender and an immigrant status could not be elaborated, nor could we investigate aspects of living in overcrowded housing. Although we aimed to achieve an even representation, no union representatives for pizza bakers took part in the study, and the three cleaners all worked at hospitals. The three union cleaners, however, all worked as general cleaners.

Strengths
This study has several strengths. We recruited participants who were exposed to potential infection risks of COVID-19, but who were part of sociodemographic groups that usually do

For peer review only - http://bmjopen.bmj.com/site/about/guidelines.xhtml

not participate in scientific studies (33). This was mostly possible because of the involvement of the health guides in the project who, through their work, had a broad trust-based social network and were able to conduct several of the interviews in the participants' native language. Since relatively little is known about the work hazards or exposure risks of these groups in the private life, it is also an advantage that we have used qualitative methodology, which is an effective tool to explore new perspectives, as it is easier to capture nuances and delve into new themes with follow-up questions. We also interviewed both workers and union representatives, through which we received broader perspectives from the latter. Additionally, the workers were interviewed over a long period of time, and all union representatives were interviewed from February to March 2021, which meant they could contribute with knowledge from different phases of the pandemic.

Conclusions

In an increasingly globalised world, pandemics are likely to re-occur. Thus, there is a need to protect all member of society equally. This interview study highlights a number of potential risks for exposure to the COVID-19 virus and lack of protection possibilities, in jobs outside the health and medical care sector dominated by foreign-born workers, as well as problems with the lack of adapted information. Our results are transferable to similar settings and can be tried in adapted actions for future infection control and other public health interventions or contribute to the themes to be explored in in larger in-depth studies in the future.

Funding

This study was funded by the Swedish research council for heath, working life and welfare (grant number: 2021-00326) and by The Swedish Research Council (grant numbers: 2020-05792, 2021-06525).

Competing interests

None

Data availability statement

Data are not publicly available. Anonymized data are available upon reasonable request from the first author.

Ethics statements

Patient consent for publication. This study was approved by the Swedish Ethics Review Authority (2021-00177).

Author contributions

MS, MM, KJ and AR conceived the study, and were part of the overall conception and design of the study. MS coordinated the data collection and drafted the manuscript. MM and JS contributed to the data collection. All authors participated in the analysis of the data and critical revision of the manuscript. All authors have read and approved the final manuscript.

References

1. Khanijahani A, Iezadi S, Gholipour K, Azami-Aghdash S, Naghibi D. A systematic review of racial/ethnic and socioeconomic disparities in COVID-19. International journal for equity in health. 2021;20(1):1-30.

For peer review only - http://bmjopen.bmj.com/site/about/guidelines.xhtml

2. Magesh S, John D, Li WT, Li Y, Mattingly-App A, Jain S, et al. Disparities in COVID-19 outcomes by race, ethnicity, and socioeconomic status: a systematic-review and meta-analysis. AMA network open. 2021;4(11).
3. Schultz WM, Kelli HM, Lisko JC, Varghese T, Shen J, Sandesara P, et al. Socioeconomic status and cardiovascular outcomes: challenges and interventions. 2018;137(20):2166-78.
4. Marmot M, Allen J. COVID-19: exposing and amplifying inequalities. Journal of Epidemiology and Community Health. 2020;74(9):681-2.
5. Baker MG. Who cannot work from home? Characterizing occupations facing increased risk during the COVID-19 pandemic using 2018 BLS data. Journal of Medrxiv. 2020.
6. Acke S, Couvreur S, Bramer WM, Schmickler M-N, De Schryver A, Haagsma J, et al. Global infectious disease risks associated with occupational exposure among non-healthcare workers: a systematic review of the literature. Occupational and Environmental Medicine, 2022;79(1):63-71.
7. Billingsley S, Brandén M, Aradhya S, Drefahl S, Mussino E, Andersson G. Deaths in the frontline: Occupation-specific COVID-19 mortality risks in Sweden. Report from University of Stockholm; 2020:36.
8. Folkhälsomyndigheten. Förekomst av covid-19 i olika yrkesgrupper. Bekräftade covid-19 fall i Sverige 13 mars-27 maj 2020. Östersund; 2020.
9. Lan F-Y, Wei C-F, Hsu Y-T, Christiani DC, Kales SN. Work-related COVID-19 transmission in six Asian countries/areas: A follow-up study. PlosOne. 2020;15(5).
10. Nafilyan V, Pawelek P, Ayoubkhani D, Rhodes S, Pembrey L, Matz M, et al. Occupation and COVID-19 mortality in England: a national linked data study of 14.3 million adults. Occupational and Environmental Medicine. 2022;79(7): 433-41.
11. Magnusson K, Nygård K, Methi F, Vold L, Telle K. Occupational risk of COVID-19 in the first versus second epidemic wave in Norway. Journal of Eurosurveillance. 2021;26(40).
12. Effekter som covid-19 har på sjukförsäkringen. (Effects of COVID-19 on health insurance). The Swedish Social Insurance Agency; 2022.
13. statistiska Centralbyrån. Yrkesregistret med yrkesstatistik 2017 Yrkesstrukturen I Sverige. Stockholm: Statistics Sweden; 2019.
14. Kjollesdal MK, Magnusson KJM. Occupational risk of COVID-19 by country of birth. A register-based study. Journal of public health, 2021: 1-7.
15. Mutambudzi M, Niedzwiedz CL, Macdonald EB, Leyland AH, Mair FS, Anderson JJ, et al. Occupation and risk of COVID-19: prospective cohort study of 120,621 UK Biobank participants. Journal of MedRxiv. 2020.
16. Bartelink V, Tynelius P, Walander A, Burström B, Ponce de Leon A, Nederby Öhd J, et al. Socioekonomiska faktorer och covid-19 i Stockholms län. Rapport 2020:10. Stockholm; 2020.
17. Ahonen EQ, Benavides FG, Benach J. Immigrant populations, work and health—a systematic literature review. Scandinavian Journal of Work, Environment & Health. 2007:96-104.
18. Berkman ND, Sheridan SL, Donahue KE, Halpern DJ, Crotty K. Low health literacy and health outcomes: an updated systematic review. Annals of Internal Medicine. 2011;155(2):97-107.
19. Ayala L, Bárcena-Martín E, Cantó O, Navarro C. Working Paper No. 73: 2021. Housing Inequalities in COVID-19 Lockdown Across European Countries. Vigo, Spain: EQUALITAS.
20. Justie B, Koonse T, Macias M, Ray J, Waheed S. Fast-Food Frontline: COVID-19 and Working Conditions in Los Angeles. University California Los Angeles (UCLA); 2022.

For peer review only - http://bmjopen.bmj.com/site/about/guidelines.xhtml

21. May T, Aughterson H, Fancourt D, Burton A. 'Stressed, uncomfortable, vulnerable, neglected': a qualitative study of the psychological and social impact of the COVID-19 pandemic on UK frontline keyworkers. BMJ open. 2021;11(11).
22. Malterud K. Systematic text condensation: a strategy for qualitative analysis. Scandinavian Journal of Public Health. 2012;40(8):795-805.
23. Molenaar J. The COVINFORM project - Migrants as a 'vulnerable group' in the COVID-19 pandemic: A European perspective. Migration and Health seminars 2021.
24. Hansson E, Albin M, Rasmussen M, Jakobsson KJL. Stora skillnader i överdödlighet våren 2020 utifrån födelseland. 2020;117.
25. Office For National Statistics. Coronavirus (COVID-19) related deaths by occupation, England and Wales: deaths registered between 9 March and 25 May 2020. London, Storbritannien; 2020.
26. James K, Thompson C, Chin-Bailey C, Davis KD, Nevins DH, Walters D, et al. COVID-19 related risk perception among taxi operators in Kingston and St. Andrew, Jamaica. Journal of transport. 2021;22.
27. Vingård E, Johansson B. Migration, arbetsmiljö och hälsa. En kunskapsöversikt. Uppsala: Uppsala Universitet; 2012.
28. Zhang H, Nardon L, Sears G. Migrant workers in precarious employment. Jounral of Equality, Diversity and Inclusion: An International Journal. 2021.
29. Esaiasson P, Johansson B, Ghersetti M, Sohlberg J. Kriskommunikation och segregation i en pandemi. Hur boende i utsatta områden informerade sig om coronaviruset våren 2020. Report from The Department of Journalism, Media and Communication, University of Gothenburg; 2020.
30. Spence PR, Lachlan KA, Burke JA. Differences in crisis knowledge across age, race, and socioeconomic status during Hurricane Ike: A field test and extension of the knowledge gap hypothesis. Journal of Communication Theory. 2011;21(3):261-78.
31. Sanchez-Ramirez DC, Normand K, Zhaoyun Y, Torres-Castro R. Long-term impact of COVID-19: a systematic review of the literature and meta-analysis. Biomedicines. 2021;9(8):900.
32. O'Neill R. WHO knew. how the world health organization (WHO) became a dangerous interloper on workplace health and safety and COVID-19. NEW SOLUTIONS: A Journal of Environmental and Occupational Health Policy. 2020;30(3):237-48.
33. Magnusson M. Bortfallet och folkhälsan. Rapport från konferensen" Varför svarar de inte på våra frågor?". Socialmedicinsk tidskrift. 2015;92(1):89-96.

For peer review only

For peer review only - http://bmjopen.bmj.com/site/about/guidelines.xhtml

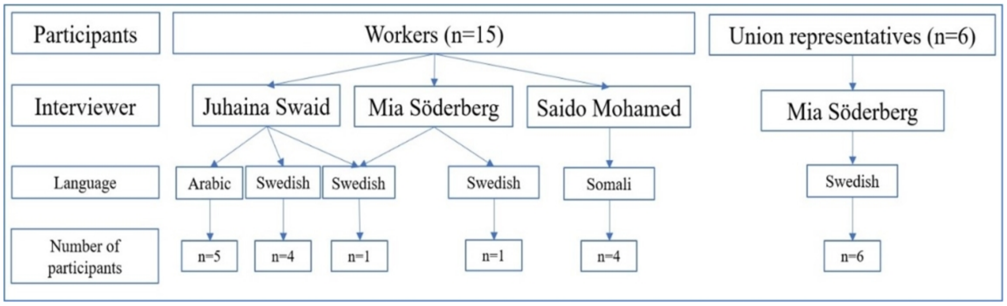

Figure 1. Languages used during interviews

162x48mm (220 x 220 DPI)

Appendix I

Descriptives of the study participants

Occupations of the workers
Tram or bus drivers (n=3)
Taxi drivers (n=3)
Property caretakers (n=2)
Hospital cleaners (n=3)
Pizza bakers (n=4)
**Union representatives and their main occupation**
Transport union: Bus drivers (n=1)
Transport union: Taxi drivers (n=1)
Property service union: Property caretaker (n=1)
Property service union: General cleaners (n=3)
**Country of origin – workers**
Turkey (n=2)
Syria (n=4)
Iraq (n=2)
Palestine (n=2)
Somalia (n=5)
**Country of origin – Union representatives**
Sweden (n=3)
Norway (n=1)
Iran (n=1)
Serbia (n=1)

For peer review only

Reporting Checklist

Item 1. Title: Concise description of the nature and topic of the study. Identifying the study as qualitative or indicating the approach (e.g., ethnography, grounded theory) or data collection methods (e.g., interview, focus group) is recommended.
Response: page 1

Item 2. Abstract: Summary of key elements of the study using the abstract format of the intended publication; typically includes background, purpose, methods, results, and conclusions.
Response: page 2

Item 3. Problem Formulation: Description and significance of the problem/phenomenon studied; review of relevant theory and empirical work; problem statement.
Response: page 3-4

Item 4. Purpose or research question: Purpose of the study and specific objectives or questions.
Response: page 4

Item 5. Qualitative approach and research paradigm: Qualitative approach (e.g., ethnography, grounded theory, case study, phenomenology, narrative research) and guiding theory if appropriate; identifying the research paradigm (e.g., post-positivist, constructivist/interpretivist) is also recommended; rationale.
Response: page 6

Item 6. Researcher characteristics and reflexivity: Researchers' characteristics that may influence the research, including personal attributes, qualifications/experience, relationship with participants, assumptions, and/or presuppositions; potential or actual interaction between researchers' characteristics and the research questions, approach, methods, results and/or transferability.
Response: page 6

Item 7. Context: Setting/site and salient contextual factors; rationale.
Response: There is no relevant guideline

Item 8. Sampling strategy: How and why research participants, documents, or events were selected; criteria for deciding when no further sampling was necessary (e.g., sampling saturation); rationale.
Response: page 4-5

Item 9. Ethical issues pertaining to human subjects: Documentation of approval by an appropriate ethics review board and participant consent, or explanation for lack thereof; other confidentiality and data security issues.
Response: page 17

Item 10. Data collection methods: Types of data collected; details of data collection procedures including (as appropriate) start and stop dates of data collection and analysis, iterative process, triangulation of sources/methods, and modification of procedures in response to evolving study findings; rationale.
Response: page 5

For peer review only

Item 11. Data collection instruments and technologies: Description of instruments (e.g., interview guides, questionnaires) and devices (e.g., audio recorders) used for data collection; if/how the instrument(s) changed over the course of the study.
Response: page 5

Item 12. Units of study: Number and relevant characteristics of participants, documents, or events included in the study; level of participation.
Response: Appendix I

Item 13. Data processing: Methods for processing data prior to and during analysis, including transcription, data entry, data management and security, verification of data integrity, data coding and anonymization / de-identification of excerpts.
Response: page 6

Item 14. Data analysis: Process by which inferences, themes, etc. were identified and developed, including the researchers involved in data analysis; usually references a specific paradigm or approach; rationale.
Response: page 6

Item 15. Techniques to enhance trustworthiness: Techniques to enhance trustworthiness and credibility of data analysis,(e.g., member checking, triangulation, audit trail); rationale.
Response: Page 6

Item 16. Synthesis and interpretation: Main findings (e.g., interpretations, inferences, and themes); might include development of a theory or model, or integration with prior research or theory.
Response: Page 7

Item 17. Links to empirical data: Evidence (e.g., quotes, field notes, text excerpts, photographs) to substantiate analytic findings.
Response: There is no relevant guideline

Item 18. Integration with prior work, implications, transferability, and contribution(s) to the field: Short summary of main findings, explanation of how findings and conclusions connect to, support, elaborate on, or challenge conclusions of earlier scholarship; discussion of scope of application/generalizability; identification of unique contribution(s) to scholarship in a discipline or field.
Response: Page 14

Item 19. Limitations: Trustworthiness and limitations of findings
Response: Page 16

Item 20. Conflicts of interest: Potential sources of influence or perceived influence on study conduct and conclusions; how these were managed.
Response: Page 17

Item 21. Funding: Sources of funding and other support; role of funders in data collection, interpretation, and reporting.
Response: Page 17

For peer review only

BMJ Open

Undervalued essential work and lacking health literacy as determinants of COVID-19 infection risks - a qualitative interview study among foreign-born workers in Sweden

Journal:	*BMJ Open*
Manuscript ID	bmjopen-2022-069838.R1
Article Type:	Original research
Date Submitted by the Author:	07-Sep-2023
Complete List of Authors:	Söderberg, Mia; University of Gothenburg, Occupational and Environmental Medicine<br>Magnusson, Maria; Angered Hospital<br>Swaid, Juhaina; University of Gothenburg, School of Public Health and Community Medicine<br>Jakobsson, Kristina; Goteborgs Universitet<br>Rosengren, Annika ; University of Gothenburg, Department of Molecular and Clinical Medicine
**Primary Subject Heading**:	Occupational and environmental medicine
Secondary Subject Heading:	Occupational and environmental medicine
Keywords:	COVID-19, OCCUPATIONAL & INDUSTRIAL MEDICINE, EPIDEMIOLOGY

SCHOLARONE™
Manuscripts

I, the Submitting Author has the right to grant and does grant on behalf of all authors of the Work (as defined in the below author licence), an exclusive licence and/or a non-exclusive licence for contributions from authors who are: i) UK Crown employees; ii) where BMJ has agreed a CC-BY licence shall apply, and/or iii) in accordance with the terms applicable for US Federal Government officers or employees acting as part of their official duties; on a worldwide, perpetual, irrevocable, royalty-free basis to BMJ Publishing Group Ltd ("BMJ") its licensees and where the relevant Journal is co-owned by BMJ to the co-owners of the Journal, to publish the Work in this journal and any other BMJ products and to exploit all rights, as set out in our licence.

The Submitting Author accepts and understands that any supply made under these terms is made by BMJ to the Submitting Author unless you are acting as an employee on behalf of your employer or a postgraduate student of an affiliated institution which is paying any applicable article publishing charge ("APC") for Open Access articles. Where the Submitting Author wishes to make the Work available on an Open Access basis (and intends to pay the relevant APC), the terms of reuse of such Open Access shall be governed by a Creative Commons licence – details of these licences and which Creative Commons licence will apply to this Work are set out in our licence referred to above.

Other than as permitted in any relevant BMJ Author's Self Archiving Policies, I confirm this Work has not been accepted for publication elsewhere, is not being considered for publication elsewhere and does not duplicate material already published. I confirm all authors consent to publication of this Work and authorise the granting of this licence.

For peer review only

Undervalued essential work and lacking health literacy as determinants of COVID-19 infection risks - a qualitative interview study among foreign-born workers in Sweden

Mia Söderberg[1], Maria Magnusson[2], Juhaina Swaid[1], Kristina Jakobsson[1,3], Annika Rosengren[4]

[1]Occupational and Environmental Medicine, School of Public Health and Community Medicine, Sahlgrenska Academy, University of Gothenburg, Sweden
[2]Angered Hospital, Hospitals West, Region Västra Götaland, Sweden
[3]Occupational and Environmental Medicine, Sahlgrenska University Hospital, Region Västra Götaland
[4]Department of Molecular and Clinical Medicine, Sahlgrenska Academy, University of Gothenburg, Sweden

Corresponding author: Mia Söderberg, Occupational and Environmental Medicine, Box 414, 40530 Gothenburg, Sweden.
Email: mia.soderberg@amm.gu.se

For peer review only

For peer review only - http://bmjopen.bmj.com/site/about/guidelines.xhtml

Abstract

Objectives: To investigate work and living conditions as determinants of COVID-19 infection risks in foreign-born workers in non-healthcare occupations.
Design: Data were collected according to a qualitative design, using semi-structured interviews. Verbatim transcripts of these interviews were analysed according to systematic text condensation.
Participants: We recruited foreign-born workers (n=15) and union representatives (n=6) in the following occupations: taxi drivers, bus and tram drivers, pizza bakers, cleaners, and property caretakers.
Results: Four overarching themes were found: "Virus exposure at work", "Aspects of low status and undervalued work", "Lack of access to information" and "Foreign-born persons' position". A notable source of virus exposure was frequent social interactions over a workday, out of which several were physically close, sometimes to the point of touching. The respondents fulfilled important societal functions, but their work was undervalued due to low job status, and they had little influence on improving safety at work. Lack of health literacy limited foreign-born workers to access information about COVID-19 infection risks and protection, since most information from employers and health organisations was only available in Swedish or disseminated through unknown channels and not adapted to their living conditions. Instead, many turned to personal contacts or social media through which a lot of misinformation was spread. Foreign-born persons were also subjected to exploitation since a Swedish residency permit could depend on maintaining employment, making it almost impossible to make demands for improved safety at work.
Conclusions: Structural factors and lack of adapted information manifested themselves as fewer possibilities for protection against COVID-19. In a globalized world, new widespread diseases are likely to occur, and more knowledge is needed to protect all workers equally. Our results are transferable to similar contexts and bring forth aspects that can be tried in quantitative studies or public health interventions.

Strengths and limitations of this study

- This study recruited participants from an already vulnerable sociodemographic group that needed tailored public health interventions during the pandemic. However, since persons from this sub-population tend to not participate in scientific studies, little was known about their particular needs.
- The use of qualitative methodology increased the possibilities of exploring new themes and extracting in-depth information about such themes.
- The interviews were mostly conducted in the participant's native language, but some interviews were carried out in a language that was neither the respondent's nor the interviewer's native language, which likely restricted a deeper understanding.
- Those who volunteered may have consisted of a selection of more well-established persons in Swedish society and almost all the participants were men.

For peer review only - http://bmjopen.bmj.com/site/about/guidelines.xhtml

Background

SARS-CoV-2 is an RNA virus that causes COVID-19, which had a pandemic spread over winter and spring 2020, reflected in high morbidity and mortality worldwide. When the World Health Organisation declared the outbreak as a pandemic (11th of March 2020), the Swedish Public Health Agency made formal requests to impose social restrictions, such as a maximum of people at social gatherings, that elderly people (>70 years) should avoid close social contacts, no outside visitors at elderly homes and that the teaching of upper secondary school pupils and university students should be done remotely. Otherwise, Swedish society remained open, with no curfews, and with shops, restaurants and elementary schools mostly operating as usual. In September 2020 when the prevalence of COVID cases was low most recommendations were relaxed, but then re-introduced in December 2020 when the numbers of cases increased rapidly. This meant stricter regulations for the allowed number of people in shops and social venues, earlier closing times for bars and restaurants, and recommendations to wear facemasks when travelling with public transport, although the latter was not mandatory.

In December 2020, the Swedish COVID-19 vaccination program started. The first batches were aimed at health and social care staff, the elderly, or those who were considered as risk groups due to a diagnosis such as cardiovascular diseases or diabetes. In June 2021, all adults ≥18 years were eligible for vaccination, which was expanded to everyone aged 16 years or older in July of the same year. The vaccination program was deemed successful as over 70% of the Swedish adult population has received at least one dose as of July 2021. As a result, almost all regulations were removed but were once again reinforced in December 2021 when the number of confirmed cases increased. Finally, most recommendations were removed in February 2022 and in March 2022 when COVID-19 no longer was considered a public and societal dangerous disease, the Swedish public health agency directed their main focus to increase vaccination rates and vaccination booster jabs.

Due to the absence of any treatment or vaccine during the first waves of the outbreak, research was foremost concentrated on clinical and pharmaceutical aspects. Yet, other public health concerns emerged as markers of a low socioeconomic position or belonging to an ethnic minority were associated with an increased risk of infection, severe illness, and death from COVID-19, increasing already existing health disparities between social strata (1, 2). Severe disease and death relate to on a higher prevalence of underlying diseases (3). Regarding infection risks, the number of social contacts is an important determinator since COVID-19 is primarily transmitted through drops in sneezing, coughing, speech, or aerosol transmission, with the latter being exacerbated in crowded and poorly ventilated spaces. In people of working age, work is a dominant source of social contacts, but with different possibilities to protect oneself. Low-wage workers tend to have fewer possibilities to practice social distancing due to the nature of their work tasks, fewer opportunities to work from home, or needing to use public transport since they lack the economical means to own a car (4, 5).

When COVID-19 was declared a pandemic, healthcare and social care occupations were promptly identified as risk occupations for becoming infected, but there were other jobs with frequent and close physical interactions, in which virus exposure and protection possibilities have been less investigated despite high COVID-19 infection rates. One review study, based on studies from the early stages of the pandemic, showed increased risks of contracting the coronavirus among abattoir workers and related food workers, and retail staff (6). Two Swedish register studies, investigating occupations outside the health and social care sector,

For peer review only - http://bmjopen.bmj.com/site/about/guidelines.xhtml

showed the highest prevalence of a positive Polymerase Chain Reaction (PCR) test for COVID-19 or death in COVID-19 (until May or June 2020) among taxi drivers, pizza bakers, and bus and tram drivers and a high prevalence among property caretakers and cleaners (7, 8). Another study, recording COVID-19 cases over the first 40 days since the first detected case in six Asian countries, noted the highest infection rates in taxi and van drivers and, in descending order, shop salespersons, religious professionals, and construction labourers (9).

As time progressed, similar and new at-risk occupations were observed. A study with access to national register data until December 2020, showed the highest COVID-19 mortality rates in taxi drivers (Hazard ratios (HR) 2.01, 95% confidence intervals (CIs) 1.67–2.43, in men), followed by support staff, bus and coach drivers, sanitary workers and van drivers, adjusted for age, multiple sociodemographic factors and prior diagnoses (10). In another national register study, the highest odds ratios (OR) for COVID-19 (a positive PCR test or an ICD-10 diagnostic code U07.1) over winter–spring 2020 was found in bus and tram drivers, taxi drivers, food counter attendants, bartenders, shops assistants and cleaners (11). During the winter of 2021, which constituted the second pandemic wave, public transport conductors, bartenders, travel stewards, waiters, food counter attendants, bus and tram drivers, taxi drivers, and hairdressers displayed the highest COVID infection rates (11). A report from the Swedish Social Insurance Agency, using sick leave data up until March 2021, illustrated a 42% increased relative risk (RR) of long-term sick leave (>21 days) in taxi drivers, compared to all other employed persons (12). Bus and tram drivers also displayed higher rates of both short-term sick leave (42% increased risk of at least one day of sick leave) and long-term sick leave (39% increased risk of >21 sick leave days).

Despite the observations of an increased risk of COVID-19 in several occupations with similar characteristics, little is known about infection risks in day-to-day situations as reported by the workers themselves. An interview study with fast-food workers (n=15) provided some insight, such as customers refusing to wear facemasks or subjecting workers to verbal abuse or threats when they were being asked to practice social distancing or to wear a facemask (13). Another study based on interviews with bus drivers, delivery workers, and police staff, illustrated late or a lack of access to protective equipment and difficulties in distancing themselves from other people (14).

Higher risks of becoming infected may also interplay with having an immigrant background as most of the Swedish occupations with the highest occurrence of COVID-19 also employ the largest proportion of foreign-born workers: pizza bakers (79%), cleaners (57%), bus and tram drivers (48%) and taxi drivers (47%) (15). Analyses based on Norwegian national register data found that workers from Somalia, Pakistan, Iraq, Afghanistan, and Turkey displayed substantially higher ORs of a COVID-19 diagnosis than the general population, in particular bus- and tram drivers (OR 12.72, 95% CI 8.29-19.55), cleaners (OR 8.81, 95% CI 6.66-11.66) and taxi drivers (OR 6.56, 95% CI 4.88-8.86), age and sex-adjusted (16). Yet it was unclear if this was related to occupational risks or other factors, as foreign-born persons in the listed occupations mostly did not display higher ORs than other immigrants from the same country. Similarly, in a British registry study, the highest mortality from COVID-19 was found among taxi drivers, bus drivers, and cleaners, but the risk estimates decreased markedly (taxi drivers and cleaners) or were nullified (bus drivers) when adjusting for age, ethnicity, housing, living in a socio-economically vulnerable area and relevant diseases (17).

The reasons for increased infection in foreign-born workers are not clear. Besides virus exposure at work, one aspect that has been brought forth is the lack of access to crisis

For peer review only - http://bmjopen.bmj.com/site/about/guidelines.xhtml

information, as well as information on safety and rights at work (18, 19). Such barriers relate to "health literacy", a concept that can be briefly explained as the use of information to protect and improve health (20). From an individual perspective, the basic level involves having adequate language skills to consume and understand information correctly, while more complex features reflect the ability to acquire relevant information and how to apply it. Health literacy also reflects organisations' ability to adapt appropriate support for health-promoting behaviour. Another potential risk factor is poor housing conditions, which is more commonly found among foreign-born persons. Sweden displays some of the largest within-country gaps in inequalities in housing deprivation in Europe, as around 30% of all foreign-born persons live in overcrowded households, compared to 9% of Swedish-born persons (21). Overcrowding in itself contributes to the spread of infection, and if co-habitants in such households work in risk occupations for COVID-19, it can exacerbate a circular infection pattern between the work and home environment.

Increased COVID-19 infection rates have been found in many occupations outside the health and social care sector, and foreign-born workers in these jobs seem to be at particular risk. Based on existing evidence it can be assumed that increased infection relates to difficulties in social distancing, as well as barriers to accessing crisis information. Yet, exposure risks in the everyday situation as described by foreign-born workers themselves are largely unknown, partly because these occupations have received less attention but also since this sociodemographic group tend to be less prone to participate in scientific studies (22). This explorative study aims to increase the understanding of pathways for COVID-19 infection in foreign-born workers in non-healthcare occupations. Based on official statistics from the Public Health Agency of Sweden we chose to investigate the study objectives in pizza bakers, taxi drivers, bus and tram drivers, cleaners, and property caretakers.

Methods

Recruitment of participants

The participants were recruited through convenience sampling, a non-random sampling method in which participants are invited through existing social networks. Initially, we only recruited foreign-born workers, but as the project progressed, we decided to include union representatives, who could contribute with an overview of the selected occupations in addition to their own experience of the working conditions. The union representatives did not have to be foreign-born. Two health guides from the East Gothenburg Health Centre were contracted for the recruitment: Juhaina Swaid (JS) (co-author) and Saido Mohamed (SM). In Sweden, health guides have a broad range of tasks such as facilitating communication between organisations and citizens, disseminating knowledge about how people can promote their health, and navigating the healthcare system to ensure that everyone in society has access to the same information. Through their work, JS and SM had a wide network of social contacts and an established trust with the people in their catchment areas. They could also conduct the interviews in several of the participants' native languages.

In all, the participants in this study were recruited through 1) social contacts of the health guides JS and SM (n=13), and 2) social contacts of colleagues at the Department of Occupational and Environmental Medicine, Sahlgrenska University Hospital (n=2). The union representatives (n=6) were recruited by 3) emailing the unions' contact persons listed on the website for each union. When contact was established, the person in question sent an invitation email to either a selection of union workers they thought could be interested in participating or to a global email list. In Sweden, most pizza bakers are self-employed or

For peer review only - http://bmjopen.bmj.com/site/about/guidelines.xhtml

work in small family-owned businesses, and we were unable to find any union representatives for this occupation. The lack of union representatives for this restaurant workers' branch was confirmed by the Swedish Hotel and Restaurant Workers' Union.

Patient and public involvement
No patients were involved in this study.

Data collection
The workers were interviewed during March 2021–March 2022, and the union representatives were interviewed in February–March 2022. To encourage the participants to freely describe their situation the interviews were carried out according to an explorative and semi-structured methodology with sets of both open-ended questions and specific questions about the work environment. The interviews were carried out by first author Mia Söderberg (MS), and the two health guides JS and SM. MS had previous experience in collecting data according to qualitative methodology. JS and SM received supervision from MS on how to conduct the interviews. JS also participated as an observer during one of the interviews that MS conducted, but this was not possible for SM. Most of the interviews took place at the participant's workplace or by Zoom or telephone. A few of the interviews were carried out at the homes of the participants or a café in their neighbourhood. The reason for the different venues was to accommodate the wishes of the participants, out of which several were hesitant to participate, or worked long hours and had many responsibilities at home, and therefore requested to be interviewed by phone or Zoom, or near their homes in an environment where they felt comfortable.

The interview guide was formulated around of three main topics: 1) virus exposure and protective measures at work, 2) access to information, and 3) living conditions. Sample of interview questions are: (virus exposure and protective measures) "How often do social contacts at work require other people to stand very close to you and shout to overcome the surrounding noise?", "What methods have you used to protect yourself from exposure to infection?", (access to information): "How do you obtain information about COVID-19?", (living conditions): "Are there any reasons why you would not call in sick if you had COVID-19 symptoms?". We also included an open question: "What do you think is the main reason for the spread of COVID-19?". The union representatives were asked the same questions both from the perspective of their own work conditions and the perspective of the members they represented. Both JS and SM had encountered some of the workers prior to this study through their work as health guides. For that reason, the interviewers took particular care to explain that the interviews and handling of any sensitive information were conducted according to professional secrecy. None of the other authors had prior contact with the participants. After the 15th interview with the workers, the content became repetitive and the data was perceived as saturated, therefore the recruitment ended. All union representatives who reported an interest in participating were interviewed.

Language considerations and transcription
The interviews were carried out by JS (native language Arabic), Saido Mohamed (SM) (native language Somali), and Mia Söderberg (MS) (native language Swedish). The languages of each interviewer are shown in Figure 1.

Figure 1. Languages used during the interviews.

For peer review only - http://bmjopen.bmj.com/site/about/guidelines.xhtml

None of the fifteen foreign-born workers had Swedish as their native language. Five of these interviews were conducted in Arabic by JS and four interviews in Somali by SM, i.e., an interview situation in the native language of both persons. The remaining six workers were interviewed in Swedish, of which four were conducted by JS, i.e., in a language that was neither the interviewer's nor the respondent's native language. With this in mind, JS revised the transcript of these interviews to evaluate if it corresponded with her perception of what emerged during the interviews and to clarify aspects that may not be apparent. The six union representatives were interviewed in Swedish by MS. Swedish was not the native language of three of the union representatives, but their language level was perceived as either equal to a native Swede (native language: Norwegian) or as an advanced Swedish speaker (native languages: Serbian and Persian). The interviews in Arabic and Somali were translated into Swedish by professional translators and all 21 interviews were then transcribed verbatim by the same company that translated the interviews. After this process, JS and SM contacted a few participants again to get further information on unclear content.

Analyses

The analysis of the data was primarily carried out by first author MS and second author Maria Magnusson (MM), who were both experienced in carrying out qualitative studies. MM has particular experience with systematic text condensation as proposed by Malterud (23), a descriptive and explorative method, which was the chosen analysis method of this study. This methodology is also characterised by paying particular attention to the participants' experiences and perspectives while emphasising an understanding of the underlying processes. MS and MM started by reading the entire transcript of all the interviews, without singling out meaningful information to get the overall gist of the material. Both authors then re-read the printed transcripts and marked all content that was perceived as meaning-bearing units with a focus on the study objectives. Other content was marked to be re-evaluated later.

In the next step, the marked units were revised and labelled into either relating to work, general living conditions, information access, or aspects of being foreign-born. From this step onwards, JS participated in the analysis process with interpretations and her professional and cultural knowledge. The themes were then further refined and presented for discussion among all the authors. The senior researchers, Kristina Jakobsson (KJ) and Annika Rosengren (AR) contributed with their expertise in public health and occupational research. The result was then edited and condensed into main themes with underlying sub-themes. The sub-themes were also evaluated with consideration to how often a certain theme was mentioned, or if the union representatives reported this as a common occurrence among the members they represented.

Results

Fifteen workers aged 30-50 were interviewed, and a majority were men (n=13). The workers were pizza bakers (n=4), property caretakers (n=2), taxi drivers (n=3), tram or bus drivers (n=3), and cleaners (n=3), out of which two worked as hospital cleaners. Time in their current occupation ranged from two to sixteen years. Six union representatives participated, three men and three women, representing property caretakers (n=1), cleaners (n=3), taxi drivers (n=1) and bus drivers (n=1). The union representatives worked with union assignments one to four days per week, the remaining time they worked in their main occupation. The number of unionised members they represented ranged from 300 to 7000 members. The countries of origin of the participants were Turkey, Syria, Iraq, Palestine, Serbia, Somalia, Norway, Iran and Sweden. All the Swedish born participants were union representatives. Most of the

For peer review only - http://bmjopen.bmj.com/site/about/guidelines.xhtml

participants lived together with their spouses and children. Further descriptive information about the study participants can be found in Appendix I.

Our analyses resulted in four overarching themes: 1. Virus exposure at work, 2. Aspects of low status and undervalued work 3. Lack of access to information and 4. Foreign-born persons' position. The main and sub-themes are presented in Table 1.

Table 1. Main and sub-themes

**Main Themes**	**Sub-themes**	
1. Virus exposure at work	1.1 Hard-to-avoid contamination risks	Social contacts<br>Surfaces
	1.2 Lack of safety culture	Employees<br>Employers<br>Employer liability
	1.3 Contributing to the spread of the disease	Social contacts over many venues<br>Social contact with vulnerable persons
2. Aspects of low status and undervalued work	2.1 Fulfilling an unappreciated societal role	Increased need for cleaning during the pandemic<br>Upholding the public transport system
	2.2 Occupational status influences workers' value and safety	Protective interventions deemed too expensive<br>Unequal protection
	2.3 Low authority over their work environment	Lack of influence<br>Distance to decision-makers
3. Lack of access to information	3.1 Inadequate health literacy	Workers<br>Employers<br>Organisations
	3.2 Consequences	Turned to unofficial information sources<br>Spread of misinformation
	3.3 Suggestions for improvements	Information in many languages<br>Utilizing complementary information outlets<br>Audio-based information
4. Foreign-born persons' position	4.1 Exploitation of a vulnerable Position	Need the job for a Swedish residency<br>Need to financially support others<br>Unawareness of regulations or rights
	4.2 Protective directives not adapted for persons outside the majority society	Cannot work from home<br>Cannot avoid public transport<br>Different social patterns

1. Virus exposure at work

Due to the nature of the work tasks and the organisational environment, the participants reported that it was nearly impossible to avoid being exposed to infection risks. Sub-themes

For peer review only - http://bmjopen.bmj.com/site/about/guidelines.xhtml

for “Virus exposure at work” were sorted into “Hard-to-avoid contamination risks”, “Lack of safety culture” and “Contributing to the spread of disease”.

1.1 Hard-to-avoid infection risks.

The most notable risk for being exposed to infection appeared to be the many social contacts the participants encountered over a workday, out of which several were physically close, and could include touching other people. For example, some bus-, tram- or taxi companies did not re-install any protective screens between the driver area and passengers during the second wave (winter 2021) and demanded that passengers enter through the front doors to facilitate ticket inspection. The drivers estimated that they would then come in close contact with more than hundreds of people in a single workday. The bus- tram and taxi drivers also carried out mobility services for citizens with reduced functional ability, such as the elderly, physically disabled persons, or school children with special needs, who often needed hands-on support.

Bus driver: "*For me, the passenger is the cause of infection. I drive you to work, to your exercise, to the store, to the health centre. One guy who stood and talked to me last week* (comment: this interview was conducted in February 2021). *He was going to the health centre for a COVID test! What the h*ll are you standing here saying, I thought*”.

Taxi driver: *“There are some who are ill. I help elderly people who can't sit down, put on their seat belts, or walk up the stairs: (Interviewer): Do you support them? (Taxi driver): Maybe I'll hold their hand, yes.”*

Taxi driver: *“Many of them need help with the seat belt and maybe help with getting into the car…(if) they have a walker - you go out and meet them, if it's slippery outside, you have to hold their arm… then you open the door and you want to hold their head so that they don't hit their head on the ceiling… yes, we touch a lot of people, even if they can manage quite well on paper. But you can't let anything happen. Say we touch 50% of all adults. I would say 80% need help with the seatbelt.”*

The participants also encountered many social interactions that were not part of their work tasks, foremost in crowded venues such as schools and shopping centres, and the hospital cleaners came into contact with patients and medical staff. Many cleaners also travelled by public transport between their daily assignments since they did not own a car, or to avoid parking fees and traffic jams, which exposed them to risks, especially since facemasks were not mandatory on public transport in Sweden. The bus and tram drivers would also use public transport to travel to the start and end stations of their shifts.

Hospital cleaner: *“The patients need help. We say no! This is not part of our job. But if they are old and can’t find the buttons* (comment: the buttons used to call for help or to operate their bed), *then we will show them the buttons. Some of the patients are old, then you have to stand close and speak loudly.”*

Hospital cleaner: *“Always when I enter this office, the doctor is sitting there. He doesn't want to leave. So, I wipe a bit there”* (comment: the participant cleans the area around where the doctor is sitting).

Union representative for cleaners: *“I have a car but I don't want to drive. You end up in traffic jams and you can't find a parking space. There’s a lot of time pressure in our work…*

For peer review only - http://bmjopen.bmj.com/site/about/guidelines.xhtml

Therefore, people are forced to go for example by subway, by bus. I can see that the people who use public transport do not use facemasks."

The participants handled tools or surfaces that had been touched by many other people, especially the property caretakers and the cleaners who operated over large areas, cleaned many different surfaces, and handled waste. The hospital cleaners rarely had the time to sanitise their equipment despite serving hospital wards, waiting rooms, and toilets.

Hospital cleaner: *In our group, we are five staff, and we use the same materials every day. (Interviewer: How often is it cleaned?) We usually take care of our materials and stuff and machines once a week. (Interviewer: You all use the same materials and the same machines?) Yes. (Interviewer: The machines and tools you use, have you increased cleaning them?) No."*

1.2 Lack of safety culture

The workers made an effort to work safely and made many requests for protective equipment at the beginning of the pandemic, but also reported that this became less important as time passed, due to stress, feeling restrained by cumbersome protective equipment, or reduced fears of the disease.

Union representative for cleaners*: "In the beginning, it was a panic about protective equipment, that's when everyone wanted to have and have and have, and finally when we got enough cleaning materials and everything they asked for that's when people started to ignore it. People eventually got used to it and said, "But nothing happens."*

Some employers introduced safety procedures and provided their workers with personal protective equipment in the early phases of the pandemic, while others seemingly delayed the provision of protective equipment and only after being pressured. The participants speculated that this was related to employers' lack of knowledge of best practices, not enough resources, or that they were unmotivated to protect their staff in a "soon-to-be-over" crisis.

Hospital cleaner: *"We got facemasks recently. The last time when corona was getting worse… they got new rules for us that we have to use facemasks* (comment: they received facemasks in October 2020)*."*

Bus driver: "*No, we didn't receive any recommendation from April to May 2020. No one warned us about this. It came after the number of infected people increased. Then the company and the municipalities began to take action and give recommendations. They started with it after the month of July* (comment: July 2020)."

Hospital cleaner: *"In the beginning, they sent us to a department called ICU and it was full of patients who had had COVID. We went there without protection, without mouth protection"* (comment: the unit was cleared of patients before cleaning). *There are so many instructions, and we know all the instructions but in the workplace, it is the opposite. It is the opposite."*

Hospital cleaner: *The emergency department built a barrack for examinations. Those who came were unwell. They had Corona or fever, or something. We asked why we are cleaning there - it's a risk! He* (comment: their manager) *said you must do it, there is no risk."*

There was also a feeling that employers only made changes that looked good "on paper" but neither cared for and sometimes even contradicted safety protocols. For example, managers

For peer review only - http://bmjopen.bmj.com/site/about/guidelines.xhtml

ignored when staff came to work with obvious COVID-19 symptoms or that lunch breaks were scheduled so that far more than eight people would be gathered in a small lunchroom that was not properly cleaned or sanitized. People who spoke up could be punished by being assigned "the graveyard shifts" or not receiving enough work to fill a full-time schedule.

Hospital cleaner: *"They say we can't have staff meetings because of being too many. When we're in the lunchroom there are more than eight people there... But I have to sit and eat!... The boss, we think he should make (changes). He doesn't!* (Comment: Eight people was the maximum allowed number of people at social gatherings at the time of the interview)".

Unclear employer liability also made it difficult to demand safer working conditions. For example, mobility services carried out by taxi drivers were often purchased by the local borough, while one company owned the vehicle, and another company might employ the driver. There was also uncertainty regarding who was responsible for the protection of cleaners against social contacts at shopping centres or in schools.

Union representative for bus drivers: "*There are several responsible persons… the one who owns the car, the company we work for, and then the one who procured the travel service - therefore there is no one to report problems to… I got frustrated and emailed the travel services committee* (comment: a committee of local politicians)*, one, two, three different persons, and got no answers*".

1.3 Contributing to the spread of the disease

Some participants worried that their work contributed to the spread of COVID-19 to other, sometimes vulnerable, people. When conducting mobility services, the taxi drivers drove up to four passengers who were sometimes picked up from crowded venues, such as community centres or schools. This caused concern as many passengers could be considered risk groups for severe COVID-19 due to old age or poor health. The hospital cleaners met both healthcare staff and patients, which meant there was a large risk of contracting and spreading the virus.

Union representative for taxi drivers: *"This is quite a large day centre… say there are 20 people, plus regular staff… and there are maybe 5-6 other residents who go to other daily activities and there are staff so if you think of the infection... I pick up this girl and then I have to pick up a regular service traveller and there is an elderly man with a facemask, and I have to put him in the back seat with her* (comment: the girl that was picked up from the service centre) *and that didn't feel good. (Interviewer: Did she have a facemask?) No no, no possibility of wearing a facemask no... it was so offensive against everyone involved."*

2) Aspects of low status and undervalued work

Despite providing important societal services there was a feeling that because they worked in what was considered low-status job, their safety and the work they performed were not considered important and valued. These aspects were sub-categorised into "Fulfilling an unappreciated societal role", "Occupational status influences workers' value and safety" and "Low authority over their work environment".

2.1 Fulfilling an unappreciated societal role

Many participants had expanded responsibilities during the pandemic and were proud of upholding important functions and contributing to the battle against COVID-19. They received appreciation for their hard work but worried that this would be forgotten once the

For peer review only - http://bmjopen.bmj.com/site/about/guidelines.xhtml

pandemic was over. There was also anger that contractors demanded extra work but did not want to offer any additional pay.

Union representative for bus drivers: *"We feel very forgotten in this. If we stop all traffic, then half of Sweden stops because then the health care workers can't reach their destination, then the teachers and students can't reach their destination. We are equal, we are not health care staff, but we are important in society."*

Union representative for cleaners: *"I think we are an important professional class, but in society, it is classified as a low professional class… We got a lot of extra orders, and they were very positive, and they were grateful for the cleaning and thanked all the cleaning companies in the community. But guaranteed, they will forget everything when the pandemic is over, unfortunately."*

2.2 Occupational status influences workers' value and safety

Many companies seemed to have treated the workers' safety as a balancing act between protecting their staff and a loss of revenue. Some transport companies did not want to install protective screens or restrict the number of allowed passengers per taxi car during the second wave (winter 2021), since that would require extra cars and staff. There were also bus companies that resisted introducing facemasks to not "scare the passengers". When facemasks or visors became mandatory this posed new problems as wearing a visor would fog-up glasses, or could cause mirror reflections, endangering driving. Some buses received a driving ban due to these mirror reflections but were put into service anyway. The union demanded restrictions from passengers entering the area near the driver, but this was met by resistance since it would make ticket inspections impossible, leading to losses in revenue.

Union representative for cleaners: *"When the first wave came, I contacted some employers to make checklists with them regarding infection (risks), they would then say, "But they already have gloves!"*

Union representative for bus drivers: "*They lost 1.6 billion (Swedish krona) last year. If you divide 1.6 billion by the number of drivers, you know the price of my life. Someone who sits in an office decides they want to gain money in this way* (comment: a loss equal to around USD 160,400,000)*."*

Union representative for taxi drivers: *"Then the infection increased and it's like, well… the front seat was closed again at the beginning of January. So, during this whole giant wave, it was open… it was awful… I called my boss and said, "I won't put anyone in the front seat". He completely panicked and said, "You can't do that", "well yes actually I can because it's about my life and health* (comment: confirmed COVID infection cases increased rapidly in November 2021, and the front seat was closed for passengers January 2022)*".*

The hospital cleaners were initially refused antigen tests at work, despite that this was freely available for the healthcare staff working on the same premises. Instead, the cleaners had to go to their local healthcare centre to get tested and were not allowed to work while waiting for the results, which meant a loss of income.

Hospital cleaner: *There is a special test at the hospital for staff there. But they sent us to our health care centre… We have to make an appointment ourselves and stay at home for four days because of this until we get an answer. Now I think it will be better… but this thing*

For peer review only - http://bmjopen.bmj.com/site/about/guidelines.xhtml

started two months ago (comment: free antigen tests were available to the cleaners at their workplace in February 2021).*"*

2.3 Low authority over their work environment
Most of the workers had limited possibilities to influence their work conditions or to make their demands and concerns heard by the management or the employers. The people who made the decisions on safety rules and other work-related aspects were far removed from the day-to-day work and had limited knowledge of the actual work content. There was also anger that those in charge worked in a safe environment from home, did not listen to workers or union representatives, and seemingly looked down on the workers.

Union representative for taxi drivers: *It's about us being just taxi drivers. Yes, and that's the hierarchy, yes… and it's really sad to say it, but that mentality is out there* (comment: among the decision makers)*, "they are dumb taxi drivers", it's impossible to get away from that."*

Union representative for bus drivers: "Y*ou sit at home, in your safe quiet living room while I'm out there driving among people and (you) talk about how I should feel at my workplace?! Damn it! Do not sit at home in your living room and think you know better than me about my workday."*

3) Lack of access to information
Throughout the interviews, it was clear that faltering health literacy resulted in a lack of access to vital information regarding infection risks and protective measures. This theme was sorted into the sub-themes "Inadequate health literacy", "Consequences" and "Suggestions for improvements".

3.1 Inadequate health literacy
Almost all participants reported that they, their colleagues, or some of the union-affiliated members they represented had insufficient language skills to properly understand crisis information in Swedish, especially if such information was formulated in a bureaucratic style, or with ambiguous phrasings. Due to a lack of information about rights even before the pandemic, many were also unsure what demand they could make regarding safety at work and provision of protective equipment when COVID-19 spread through society.

Taxi driver: *"The news reporting about the disease was in Swedish, there was no Arabic channel that gave information about it in Arabic or other languages. There are many people in Sweden who do not know Swedish. They know limited Swedish, which is not good enough."*

Pizza baker: "*What I don't understand is – there's no requirement for facemasks. And then they say that we should wear facemasks when it is… high time? Or when it is rush hour? I cannot know what time it is rush hour… should I wear a facemask or not?*"

Union representative for cleaners: *"As I said, we have a bit of a problem with the language. People might feel worried if they want to complain or report something, they don't know that they have the right to do so. They get scared and don't dare say anything. That's the lack we actually have."*

For peer review only - http://bmjopen.bmj.com/site/about/guidelines.xhtml

Health literacy is a two-sided process and involves the competency of the information disseminator, but as of winter 2022, many employers still only distribute safety information in Swedish and through arbitrary methods. Taxi, bus, and tram drivers and property caretakers, whose routes and assignments need to be coordinated, received information via staff apps or web platforms. For most others, information was spread through methods such as text messages or leaflets on the staff noticeboards. This information was mostly in Swedish, and the participants figured that their employers knew that some workers wouldn't understand, but that it "looked good on paper". Some participants received oral information at staff meetings, but this was also in Swedish.

Union representative for cleaners: *"They have an information board in the office, people go by and collect materials or gather there. But they just put information on the board, I think you should say that this information applies to Corona so go and read it for example. (Interviewer: They think they have done their employer responsibility, but they don't check that people actually read and if they understand?) No exactly."*

Bus driver*: "The Swedes are a people who trust the government, (but) the Swedish media have not explained clearly, in a good way about corona for migrants or new arrivals, the consequence of this was that a large part of them died from corona."*

Hospital cleaner: *"In our job, there are many foreigners. They don't understand the language very well. Sometimes they sit and listen* (comment: during the staff meetings) *but they don't understand anything."*

<u>*3.2 Consequences*</u>

The lack of adapted information from employers and official outlets resulted in almost none of the workers using official public health outlets as a source of information, at least not during the first stages of the pandemic. For many of them the Public Health Agency of Sweden or the health care information organization "1177", the main distributors of COVID-related information, was unknown. The workers primarily received their information from news channels in their country of origin, from friends and relatives, or social media, through which a lot of misinformation was spread. Some participants also mentioned institutions that they were familiar with and trusted, such as their local healthcare centre or pharmacist, as reliable information sources.

Taxi driver: *"At the beginning, a friend, a doctor, said that corona did not exist. Such information has confused us a bit, hasn't it? A doctor… who says that there is no such thing as corona. In the beginning, I heard many rumours, that there are many old people in Sweden that they want to get rid of, right? In the beginning, we heard a lot of contradictory information."*

Property caretaker: "*(Interviewer: 1177 have a webpage that is translated into Arabic. If something happens, would you call…?). I did not make contact with anyone because of what was spread in the media, Facebook, and from the doctors. It was different (conflicting) information. I thought it was not beneficial. (Interviewer: If you want to obtain information about COVID-19, which information source do you trust?) My relatives."*

Taxi driver: *"(Interviewer: Which source of information do you trust the most?) Ah…TV. There are many people who watch TV, and the radio as well.* (Comment: TV and radio from their home countries).*"*

For peer review only - http://bmjopen.bmj.com/site/about/guidelines.xhtml

Property caretaker: *"(Interviewer: Do you trust the authorities?) No, I don't. I am newly arrived in Sweden, there are many websites that are linked to the authorities that I am not aware of. I don't know who to contact. I might contact the health centre or the pharmacy."*

3.3 Suggestions for improvements

The respondents made several suggestions on how information dissemination could be improved, primarily emphasizing the need for information in more languages than Swedish. During times of less strict regulations, information could be disseminated at outdoor meetings. Some participants also expressed a sense of trust in health centres or pharmacies, and that these health institutions could have an expanded role in providing the local community with information. There were also suggestions that information regarding responsibilities and how to conduct safety at work could be sent by post to all people who were registered as running businesses. Since some immigrants, especially older people, have difficulties reading and writing, some also thought it would be beneficial to produce audio-based information, which could be disseminated on government websites.

Taxi driver: "My grandfather will not understand printed information if you give him this leaflet, information should be in audio format and easy to understand."

Property caretaker: *"Authorities who know that I run a business should have contacted me, they should have sent me a letter or texted me."*

4) Foreign-born persons' position

Some aspects were primarily tied to having an immigrant background and these were sorted into the sub-themes: "Exploitation of a vulnerable position" and "Protective directives not adapted for persons outside the majority".

4.1 Exploitation of a vulnerable position

One of the most salient observations from the union representatives was difficulties in improving protection in cases where employment was a prerequisite to upholding a Swedish residency permit. As told by some of the union representatives, there are several Swedish vocational training programs designed for new immigrants and therefore a high proportion of foreign-born persons without permanent residency in some occupations, which sometimes could be taken advantage of. Some employers also appeared to be aware that many immigrants need to financially support their relatives, which also might be abused, as speaking up against poor working conditions could result in fewer working hours or being assigned the worst shifts. Such aspects were also a reason for not joining the union since the union does not accept unregulated overtime.

Union representative for bus drivers: "*We often notice that newly arrived persons attend SFI* (comment: Swedish For Immigrants) *training programs and the vocational bus training program. You are then completely dependent on your boss to get a residency permit. They would rather drive and get the wrong salary to get a residence permit. Because of this, I have chosen representatives against these employers who actually choose to exploit these people. This is almost modern slavery, "you do as I say, or you go home"* (comment: she takes extra care to choose union representatives to combat these issues)".

For peer review only - http://bmjopen.bmj.com/site/about/guidelines.xhtml

Union representative taxi driver: *Many of them then want to work a lot, they want money. Many send money home, many come from vulnerable (residential) areas so their children should get the same things as other children therefore they are at a disadvantage. Our driving and rest time rules mean that they are quite limited in how much they can work. If you're in the union, you can't cheat. (Interviewer: Do the employers know about this?) Oh yes. (Interviewer: Do you think this is the case in your industry?) Yes absolutely."*

4.2 Protective directives not adapted for persons outside the majority society

The participants reported that many of the safety recommendations disseminated by the Public Health Agency of Sweden, such as working from home or avoiding public transport, could not be adapted to the participants' life situation. One new theme that arose, for which there was no adapted information, was large social gatherings with persons from the same country, often privately arranged at cafés or other local venues. Other examples were large gatherings in cramped local food stores, mostly owned by other immigrants.

Pizza baker: *"We have a cafe where you gather after work… How many were there last Friday or Saturday…I don't think there were more than 40 people.* (Comment: the interview was conducted when eight persons were the maximum number of allowed people at social gatherings).*"*

Pizza baker: *"The (local) grocery store, that's it, 1000 people who come to the grocery store, move all the time, (touch) all the stuff, the bags, so there's a risk in the grocery store."*

One aspect of interest in this study was overcrowding, but none of the interviewed persons could be defined as living in overcrowded conditions. Some of the union representatives mentioned that their members lived in overcrowded housing, but since none of the respondents themselves lived like this we could not ask for more detailed information. Only two workers and one union representative reported limited finances as an obstacle for sick leave. Otherwise, most of the respondents reasoned that one's health was more important than money or that they were financially secure enough to go on sick leave if they needed it.

Discussion

This explorative interview study resulted in four overarching themes. The first concerned virus exposure at work, mainly through large numbers of social interactions and a lack of safety culture. The second theme related to working in a society-deemed low-status occupation where the participants' work and they as workers were not adequately appreciated or protected, despite carrying out important tasks for societal function. The third theme revolved around a lack of health literacy which meant that participants had limited access to essential crisis information, and instead turned to other information channels, through which a lot of misinformation was spread, causing confusion and the use of maladapted protective behaviour. Lastly, there were risk aspects tied to being foreign-born. Most notably a precarious position in the labour market and the need to keep employment to maintain residency in Sweden, which could lead to exploitation and difficulties in speaking up against hazardous working conditions. There was also a lack of directives that were adapted to persons outside the majority society.

Virus exposure at work

Concerning virus exposure at work, the media and public health organisations have mainly focused on the health and social care sector, but other industries and large occupational

For peer review only - http://bmjopen.bmj.com/site/about/guidelines.xhtml

groups also experienced increased risks of virus exposure at work (5, 6, 7, 8). International studies have consistently found that working in a low-status occupation is associated with an increased risk of COVID-19 and in this study, we took a closer look at five Swedish occupations (7, 8). Weighing in hard-to-avoid sources of potential infection and poor organisational safety culture, cleaners, taxi, bus, and tram drivers seemed notably vulnerable to many and physically close social contacts, which may explain why infection and mortality remained high among bus, tram, and taxi drivers throughout the pandemic (11, 12). The many social contacts could also contribute to an increased spread of the disease to other people, some of whom could be considered risk groups for developing severe COVID-19.

A few studies with taxi- and public transport drivers have presented similar findings (24), while little is known about cleaners, property caretakers, and pizza bakers, with the latter perhaps being best comparable to fast-food workers from an international perspective. An American study among fast-food workers with data collected from both questionnaires (n=417) and interviews (n=15) (13) illustrated several occupational hazards. These were partly linked to employers' inadequate investment and interest in safety culture, such as not providing staff with facemasks. Forty percent of workers needed to buy their own facemasks, and out of those, around 10% of respondents were unable to afford them. Many co-workers or customers did not wear facemasks, with frequent incidents of threats and verbal abuse from customers when the staff tried to make them adhere to safety protocols (13). In contrast, the pizza bakers in our study were mostly self-employed and appeared to be very able to create protective routines that were respected by staff and customers.

Aspects of low status and undervalued work

Amid the pandemic, the inequities between different groups of workers became apparent, as workers in higher-status jobs could protect themselves, for example by working from home, while those in already precarious jobs needed to work on-site, exposing themselves to the risk of infection. In most countries, including Sweden, healthcare workers were hailed as heroes during the pandemic. However, the workers in our study, who also provide important functions for the upholding of society, did not receive the same widespread acclaim. Bus, tram, and taxi drivers sustained the transport infrastructure and cleaners had an enhanced role since increased cleaning was regarded as an important measure to prevent the spread of COVID-19. One qualitative study from the UK (14), conducted among bus drivers and transport workers, found similar themes as this study, primarily a sense of being unprotected at work, late or no introduction of protective equipment, as well as comparing themselves with other essential workers but without the same level of appreciation.

The definition of a low-status job is often related to a lack of longer formal training requirements or how work tasks are valued. Our investigated occupations can, by this definition, be viewed as low-status jobs. As such, one respondent reported that the employer calculated the financial loss for protective interventions and weighted this against the safety of their staff. The distance between the workers, decision-makers, and employers also resulted in frustration since the people in authority often lacked knowledge of their work conditions and how to create a protective work environment.

Lack of access to information

Another finding was the double-sided lack of health literacy from both the receiver's and the sender's perspectives. When COVID-19 was declared a full-scale pandemic, it dominated the news flow worldwide and awareness of the disease was high, but crucial information on what constituted a risk for infection and protective measures, did not reach everyone equally. An

For peer review only - http://bmjopen.bmj.com/site/about/guidelines.xhtml

EU-funded consortium (COVINFORM) concluded that morbidity in COVID-19 partly depended on “Vulnerability in relation to communication”, meaning lack of access to, or poorly adapted, information (25). A Swedish study conducted before the pandemic illustrated that in geographical areas marked by low socioeconomic status and with a large proportion of first- and second-generation immigrants, there is a higher use of social media, foreign news media, or social networks as the main source of information, especially among people with limited Swedish language proficiency (26). This difficulty in accessing important societal information is referred to as “the knowledge gap”, and has been observed in several countries, in which foreign-born persons have more difficulties accessing crisis information since they lack social connections with knowledge about the new country or that they distrust authorities (19).

Health literacy tends to focus on the skills of the recipient, but it is indeed a two-way process in which the disseminator, in this context, employers and the Swedish health authorities, have a responsibility for disseminating information in a manner that is both received, understood and adapted to the communication patterns of the receiver. This is not only the ethically correct approach but also has a bearing on fundamental human rights in being given the possibility to protect one’s health. To achieve this, the receivers of information should be given the opportunity to influence, by being provided with communication channels to those responsible for compiling and disseminating information. In this way, knowledge of communication channels, special needs, and unknown risks can increase, and public health interventions can be designed with better precision.

Foreign-born persons' position

International studies have nearly unanimously concluded that the risk of contracting COVID-19 is higher in immigrants in low-status jobs, a group of workers that even before the pandemic were at larger risk of work-related ill-health. A review study by the Swedish Work Environment Authority showed that individuals with an immigrant background more often worked in stressful jobs that required short formal training and in these occupations were assigned the worst tasks or received the least safety training (27). Unfortunately, people from these social groups tend to not participate in scientific studies, partly due to a lack of trust, but also since participation often requires language proficiency to, for example, fill in questionnaires. Studies based on self-reported data are then not only scarce but also often biased, reflecting the experiences of those who are more integrated into Swedish society.

One of the most alarming findings in this study was the occurrence of exploitation, as some employers appeared to abuse the fact that many immigrants are dependent on their employment to keep their Swedish residency permit. These findings correspond with studies from other countries in which immigrants, especially those newly arrived, more often work in precarious work situations due to problems entering the labour market and the dependency on their jobs to legally remain in the new country (28, 29). Furthermore, most of the occupations investigated in this study require short or no formal training and no need for high language skills, which likely means that there is a large pool of available workers and that it is easy to replace people who are perceived as difficult or demanding. Several union representatives reported that speaking up could lead to punishments such as being assigned the “graveyard shifts” or getting too few hours to fill a work week. One qualitative interview study among foreign-born cleaners in Sweden (30) illustrated a high competition between cleaning companies, but also many available workers which contributed to difficulties in receiving enough shifts to fulfil 100% employment. Due to these factors, the best shifts and a full-time schedule were often given to the workers who did not complain and who were on good terms

For peer review only - http://bmjopen.bmj.com/site/about/guidelines.xhtml

with the foremen and employers. This made it difficult, if not impossible, to demand improved work conditions. If similar tendencies exist in the occupations selected for our study, it further illustrates how the workers are exposed to large risks at work, as well as being powerless over the conditions that expose them to a potentially lethal disease. Little is still known about long-term ill-health from COVID-19, but this already vulnerable group may also face higher risks of future reduced workability from post-COVID (31). In light of such disparities, there has been a debate as to whether health organisations such as the WHO have a responsibility to enforce directives to protect workers (32).

Other considerations
This study had an explorative approach and used a set of questions with the possibility of follow-up questions to investigate the study's aims. Because of the qualitative design, external validity and generalizability cannot be tested. The participants commonly described similar occurrences of risk situations for exposure to the SARS-CoV-2 virus, and the stability of these responses indicates that our findings have transferability to similar job situations. This was further reemphasized by the interviews with union representatives that have an overview of each occupation from representing large numbers of unionized members.

Finally, there is a risk that a study on this particular topic may support a discourse of blame and stigmatisation. Even before the COVID-19 pandemic, the public debate concerning certain demographic groups and disadvantaged geographical areas was characterised by a negative and one-sided approach. Such problematic images are at risk of being exacerbated, as certain urban areas or ethnic groups displayed a higher occurrence of COVID-19, at the expense of understanding the underlying causes. It is therefore important that future studies acknowledge the existing structural inequalities to avoid that groups with limited resources to govern their life situation being blamed for increasing the spread of a disease.

Limitations
Based on the language proficiency of the interviewers and the respondents, we only conducted interviews in Swedish, Arabic, and Somali. Four interviews were conducted in a language (Swedish) that was neither the respondent's nor the interviewer's native language, which likely restricted a mutual deeper understanding. It is also likely that those who volunteered were a selection of more well-established people and with a higher level of trust in Swedish society and that we failed to recruit those most at risk. Future studies should conduct data collection in every participant's native language and preferably by someone who also is knowledgeable in the participants' culture. There should also be more efforts to facilitate trust so that people who can be defined as marginalized feel comfortable to participate.

Almost all the participants were men since most of the selected occupations are male-dominated. Nevertheless, foreign-born women more often report poorer working conditions or leaving the labour market prematurely, compared to both foreign-born men and Swedish-born women (27). Because of the male majority, aspects of dual vulnerability based on gender and immigrant status could not be elaborated, and efforts to recruit women should be made in future studies. Although we aimed to achieve an even representation, no union representatives for pizza bakers took part in the study, and the three cleaners all worked at hospitals. The three union cleaners, however, all worked as general cleaners. Also, since none of the participants could be considered as living in an overcrowded household, we could not investigate this aspect.

For peer review only - http://bmjopen.bmj.com/site/about/guidelines.xhtml

Strengths

This study has several strengths. Foremost, that we could recruit participants from sociodemographic groups that previously have been identified as most at-risk of COVID-19 infection, but who usually do not participate in scientific studies (22). The lack of knowledge of the circumstances in such groups leads to a risk that public health interventions are not designed for those who need them the most. The successful recruitment was made possible because of the contracted health guides who, through their work, had a broad trust-based social network and were able to conduct several of the interviews in the participant's native language. Since relatively little is known about the work hazards and exposure risks of these groups in their private life, it is also an advantage that we have used qualitative methodology, which is an effective tool to explore new perspectives, since it makes it possible to capture nuances and delve into new themes with follow-up questions. We also interviewed both workers and union representatives. Through the latter, we could receive a broader perspective and gain information on negotiations with employers. Additionally, all union representatives were interviewed in February and March 2021, which meant they could contribute with knowledge from different phases of the pandemic.

Conclusions

In an increasingly globalised world, pandemics are likely to reappear and when widespread diseases enter all members of society should be protected equally. This interview study highlights several potential risks for exposure to the COVID-19 virus and the lack of protection possibilities in jobs outside the health and medical care sector dominated by foreign-born workers, as well as problems with the lack of adapted information. Our results are transferable to similar contexts and bring forth aspects that can be further explored in quantitative studies and public health interventions.

Funding

This study was funded by the Swedish Research Council for Health, Working Life and welfare (grant number: 2021-00326) and by the Swedish Research Council (grant numbers: 2021-06525).

Competing interests

None

Data availability statement

Data are not publicly available. Anonymized data are available upon reasonable request from the first author and pending fulfilment of legal requirements.

Ethics statements

Patient consent for publication. This study was approved by the Swedish Ethics Review Authority (2021-00177).

Author contributions

MS, MM, KJ, and AR conceived the study and were part of the overall conception and design of the study. MS coordinated the data collection and drafted the manuscript. MM and JS

For peer review only - http://bmjopen.bmj.com/site/about/guidelines.xhtml

contributed to the data collection. All authors participated in the analysis of the data and critical revision of the manuscript. All authors have read and approved the final manuscript.

References

1. Khanijahani A, Iezadi S, Gholipour K, Azami-Aghdash S, Naghibi D. A systematic review of racial/ethnic and socioeconomic disparities in COVID-19. International journal for equity in health. 2021;20(1):1-30.
2. Magesh S, John D, Li WT, Li Y, Mattingly-App A, Jain S, et al. Disparities in COVID-19 outcomes by race, ethnicity, and socioeconomic status: a systematic review and meta-analysis. AMA network open. 2021;4(11).
3. Schultz WM, Kelli HM, Lisko JC, Varghese T, Shen J, Sandesara P, et al. Socioeconomic status and cardiovascular outcomes: challenges and interventions. 2018;137(20):2166-78.
4. Marmot M, Allen J. COVID-19: exposing and amplifying inequalities. Journal of Epidemiology and Community Health. 2020;74(9):681-2.
5. Baker MG. Who cannot work from home? Characterizing occupations facing increased risk during the COVID-19 pandemic using 2018 BLS data. Journal of Medrxiv. 2020.
6. Acke S, Couvreur S, Bramer WM, Schmickler M-N, De Schryver A, Haagsma J, et al. Global infectious disease risks associated with occupational exposure among non-healthcare workers: a systematic review of the literature. Occupational and Environmental Medicine, 2022;79(1):63-71.
7. Billingsley S, Brandén M, Aradhya S, Drefahl S, Mussino E, Andersson G. Deaths in the frontline: Occupation-specific COVID-19 mortality risks in Sweden. Report from University of Stockholm; 2020:36.
8. Public Health Agency of Sweden. Förekomst av covid-19 i olika yrkesgrupper. Bekräftade covid-19 fall i Sverige 13 mars-27 maj 2020. *(Translated: The occurrence of COVID-19 in different occupational groups. Confirmed COVID-19 cases in Sweden 13 March-27 May 2020.)* Östersund; 2020.
9. Lan F-Y, Wei C-F, Hsu Y-T, Christiani DC, Kales SN. Work-related COVID-19 transmission in six Asian countries/areas: A follow-up study. PlosOne. 2020;15(5).
10. Nafilyan V, Pawelek P, Ayoubkhani D, Rhodes S, Pembrey L, Matz M, et al. Occupation and COVID-19 mortality in England: a national linked data study of 14.3 million adults. Occupational and Environmental Medicine. 2022;79(7): 433-41.
11. Magnusson K, Nygård K, Methi F, Vold L, Telle K. Occupational risk of COVID-19 in the first versus second epidemic wave in Norway. Journal of Eurosurveillance. 2021;26(40).
12. Effekter som covid-19 har på sjukförsäkringen. *(Translated: Effects of COVID-19 on health insurance).* The Swedish Social Insurance Agency; 2022.
13. Justie B, Koonse T, Macias M, Ray J, Waheed S. Fast-Food Frontline: COVID-19 and Working Conditions in Los Angeles. University California Los Angeles (UCLA); 2022.
14. May T, Aughterson H, Fancourt D, Burton A. 'Stressed, uncomfortable, vulnerable, neglected': a qualitative study of the psychological and social impact of the COVID-19 pandemic on UK frontline keyworkers. BMJ Open. 2021;11(11).
15. Statistics Sweden (Statistiska Centralbyrån). Yrkesregistret med yrkesstatistik 2017. Yrkesstrukturen i Sverige. *(Translated: The occupational register with occupational statistics 2017. The occupational structure in Sweden.)* Stockholm: Statistics Sweden; 2019.
16. Kjollesdal MK, Magnusson KJM. Occupational risk of COVID-19 by country of birth. A register-based study. Journal of Public Health, 2021: 1-7.

For peer review only - http://bmjopen.bmj.com/site/about/guidelines.xhtml

17. Mutambudzi M, Niedzwiedz CL, Macdonald EB, Leyland AH, Mair FS, Anderson JJ, et al. Occupation and risk of COVID-19: prospective cohort study of 120,621 UK Biobank participants. Journal of MedRxiv. 2020.
18. Ahonen EQ, Benavides FG, Benach J. Immigrant populations, work and health—a systematic literature review. Scandinavian Journal of Work, Environment & Health. 2007:96-104.
19. Spence PR, Lachlan KA, Burke JA. Differences in crisis knowledge across age, race, and socioeconomic status during Hurricane Ike: A field test and extension of the knowledge gap hypothesis. Journal of Communication Theory. 2011;21(3):261-78.
20. Berkman ND, Sheridan SL, Donahue KE, Halpern DJ, Crotty K. Low health literacy and health outcomes: an updated systematic review. Annals of Internal Medicine. 2011;155(2):97-107.
21. Ayala L, Bárcena-Martín E, Cantó O, Navarro C. Working Paper No. 73: 2021. Housing Inequalities in COVID-19 Lockdown Across European Countries. Vigo, Spain: EQUALITAS.
22. Magnusson M: Bortfallet och folkhälsan. Conference report ”Varför svarar de inte på våra frågor?” (Translated: "Why don't they answer our questions?"). Socialmedicinsk tidskrift. 2015, 92(1):89-96.
23. Malterud K. Systematic text condensation: a strategy for qualitative analysis. Scandinavian Journal of Public Health. 2012;40(8):795-805.
24. James K, Thompson C, Chin-Bailey C, Davis KD, Nevins DH, Walters D, et al. COVID-19 related risk perception among taxi operators in Kingston and St. Andrew, Jamaica. Journal of transport. 2021;22.
25. Molenaar J. The COVINFORM project - Migrants as a ‘vulnerable group’ in the COVID-19 pandemic: A European perspective. Migration and Health seminars 2021.
26. Esaiasson P, Johansson B, Ghersetti M, Sohlberg J. Kriskommunikation och segregation i en pandemi. Hur boende i utsatta områden informerade sig om coronaviruset våren 2020. *(Translated: Crisis communication and segregation in a pandemic. How residents in vulnerable areas informed themselves about the coronavirus in spring 2020.)* Report from The Department of Journalism, Media and Communication, University of Gothenburg; 2020.
27. Vingård E, Johansson B. Migration, arbetsmiljö och hälsa - En kunskapsöversikt. *(Translation: Migration, work environment and health – a literature overview)*. Rapport 2012:4. The Swedish Work Environment Authority. Uppsala: Uppsala Universitet; 2012.
28. Zhang H, Nardon L, Sears G. Migrant workers in precarious employment. Journal of Equality, Diversity and Inclusion: An International Journal. 2021.
29. Anderson B. Migration, immigration controls and the fashioning of precarious workers. Work, employment society. 2010;24(2):300-17.
30. Abbasian S, Hellgren C. Working conditions for female and immigrant cleaners in Stockholm County: An intersectional approach. Nordic journal of working life studies. 2012;2(3):161-81.
31. Sanchez-Ramirez DC, Normand K, Zhaoyun Y, Torres-Castro R. Long-term impact of COVID-19: a systematic review of the literature and meta-analysis. Biomedicines. 2021;9(8):900.
32. O’Neill R. WHO knew. How the world health organization (WHO) became a dangerous interloper on workplace health and safety and COVID-19. NEW SOLUTIONS: A Journal of Environmental and Occupational Health Policy. 2020;30(3):237-48.

For peer review only - http://bmjopen.bmj.com/site/about/guidelines.xhtml

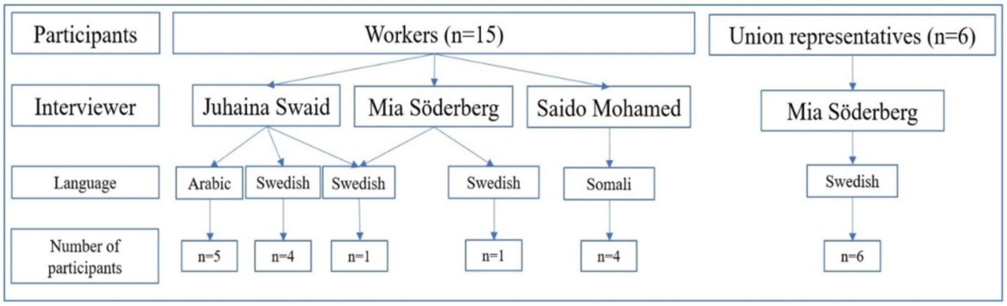

Figure 1. Languages used during interviews

162x48mm (220 x 220 DPI)

Appendix I

Descriptives of the study participants

Occupations of the workers
Tram or bus drivers (n=3)
Taxi drivers (n=3)
Property caretakers (n=2)
Cleaners (n=3)
Pizza bakers (n=4)
**Union representatives and their main occupation**
Transport union: Bus drivers (n=1)
Transport union: Taxi drivers (n=1)
Property service union: Property caretaker (n=1)
Property service union: General cleaners (n=3)
**Country of origin – workers**
Turkey (n=2)
Syria (n=4)
Iraq (n=2)
Palestine (n=2)
Somalia (n=5)
**Country of origin – Union representatives**
Sweden (n=3)
Norway (n=1)
Iran (n=1)
Serbia (n=1)

For peer review only

Reporting Checklist

Item 1. Title: Concise description of the nature and topic of the study. Identifying the study as qualitative or indicating the approach (e.g., ethnography, grounded theory) or data collection methods (e.g., interview, focus group) is recommended.
Response: page 1

Item 2. Abstract: Summary of key elements of the study using the abstract format of the intended publication; typically includes background, purpose, methods, results, and conclusions.
Response: page 2

Item 3. Problem Formulation: Description and significance of the problem/phenomenon studied; review of relevant theory and empirical work; problem statement.
Response: page 3-4

Item 4. Purpose or research question: Purpose of the study and specific objectives or questions.
Response: page 4

Item 5. Qualitative approach and research paradigm: Qualitative approach (e.g., ethnography, grounded theory, case study, phenomenology, narrative research) and guiding theory if appropriate; identifying the research paradigm (e.g., post-positivist, constructivist/interpretivist) is also recommended; rationale.
Response: page 6

Item 6. Researcher characteristics and reflexivity: Researchers' characteristics that may influence the research, including personal attributes, qualifications/experience, relationship with participants, assumptions, and/or presuppositions; potential or actual interaction between researchers' characteristics and the research questions, approach, methods, results and/or transferability.
Response: page 6

Item 7. Context: Setting/site and salient contextual factors; rationale.
Response: There is no relevant guideline

Item 8. Sampling strategy: How and why research participants, documents, or events were selected; criteria for deciding when no further sampling was necessary (e.g., sampling saturation); rationale.
Response: page 4-5

Item 9. Ethical issues pertaining to human subjects: Documentation of approval by an appropriate ethics review board and participant consent, or explanation for lack thereof; other confidentiality and data security issues.
Response: page 17

Item 10. Data collection methods: Types of data collected; details of data collection procedures including (as appropriate) start and stop dates of data collection and analysis, iterative process, triangulation of sources/methods, and modification of procedures in response to evolving study findings; rationale.
Response: page 5

For peer review only

Item 11. Data collection instruments and technologies: Description of instruments (e.g., interview guides, questionnaires) and devices (e.g., audio recorders) used for data collection; if/how the instrument(s) changed over the course of the study.
Response: page 5

Item 12. Units of study: Number and relevant characteristics of participants, documents, or events included in the study; level of participation.
Response: Appendix I

Item 13. Data processing: Methods for processing data prior to and during analysis, including transcription, data entry, data management and security, verification of data integrity, data coding and anonymization / de-identification of excerpts.
Response: page 6

Item 14. Data analysis: Process by which inferences, themes, etc. were identified and developed, including the researchers involved in data analysis; usually references a specific paradigm or approach; rationale.
Response: page 6

Item 15. Techniques to enhance trustworthiness: Techniques to enhance trustworthiness and credibility of data analysis,(e.g., member checking, triangulation, audit trail); rationale.
Response: Page 6

Item 16. Synthesis and interpretation: Main findings (e.g., interpretations, inferences, and themes); might include development of a theory or model, or integration with prior research or theory.
Response: Page 7

Item 17. Links to empirical data: Evidence (e.g., quotes, field notes, text excerpts, photographs) to substantiate analytic findings.
Response: There is no relevant guideline

Item 18. Integration with prior work, implications, transferability, and contribution(s) to the field: Short summary of main findings, explanation of how findings and conclusions connect to, support, elaborate on, or challenge conclusions of earlier scholarship; discussion of scope of application/generalizability; identification of unique contribution(s) to scholarship in a discipline or field.
Response: Page 14

Item 19. Limitations: Trustworthiness and limitations of findings
Response: Page 16

Item 20. Conflicts of interest: Potential sources of influence or perceived influence on study conduct and conclusions; how these were managed.
Response: Page 17

Item 21. Funding: Sources of funding and other support; role of funders in data collection, interpretation, and reporting.
Response: Page 17

For peer review only

BMJ Open

Undervalued essential work and lacking health literacy as determinants of COVID-19 infection risks - a qualitative interview study among foreign-born workers in Sweden

Journal:	*BMJ Open*
Manuscript ID	bmjopen-2022-069838.R2
Article Type:	Original research
Date Submitted by the Author:	23-Nov-2023
Complete List of Authors:	Söderberg, Mia; University of Gothenburg, Occupational and Environmental Medicine<br>Magnusson, Maria; Angered Hospital<br>Swaid, Juhaina; University of Gothenburg, School of Public Health and Community Medicine<br>Jakobsson, Kristina; Goteborgs Universitet<br>Rosengren, Annika ; University of Gothenburg, Department of Molecular and Clinical Medicine
**Primary Subject Heading**:	Occupational and environmental medicine
Secondary Subject Heading:	Occupational and environmental medicine
Keywords:	COVID-19, OCCUPATIONAL & INDUSTRIAL MEDICINE, EPIDEMIOLOGY

SCHOLARONE™
Manuscripts

I, the Submitting Author has the right to grant and does grant on behalf of all authors of the Work (as defined in the below author licence), an exclusive licence and/or a non-exclusive licence for contributions from authors who are: i) UK Crown employees; ii) where BMJ has agreed a CC-BY licence shall apply, and/or iii) in accordance with the terms applicable for US Federal Government officers or employees acting as part of their official duties; on a worldwide, perpetual, irrevocable, royalty-free basis to BMJ Publishing Group Ltd ("BMJ") its licensees and where the relevant Journal is co-owned by BMJ to the co-owners of the Journal, to publish the Work in this journal and any other BMJ products and to exploit all rights, as set out in our licence.

The Submitting Author accepts and understands that any supply made under these terms is made by BMJ to the Submitting Author unless you are acting as an employee on behalf of your employer or a postgraduate student of an affiliated institution which is paying any applicable article publishing charge ("APC") for Open Access articles. Where the Submitting Author wishes to make the Work available on an Open Access basis (and intends to pay the relevant APC), the terms of reuse of such Open Access shall be governed by a Creative Commons licence – details of these licences and which Creative Commons licence will apply to this Work are set out in our licence referred to above.

Other than as permitted in any relevant BMJ Author's Self Archiving Policies, I confirm this Work has not been accepted for publication elsewhere, is not being considered for publication elsewhere and does not duplicate material already published. I confirm all authors consent to publication of this Work and authorise the granting of this licence.

For peer review only

Undervalued essential work and lacking health literacy as determinants of COVID-19 infection risks - a qualitative interview study among foreign-born workers in Sweden

Mia Söderberg[1], Maria Magnusson[2], Juhaina Swaid[1], Kristina Jakobsson[1,3], Annika Rosengren[4]

[1]Occupational and Environmental Medicine, School of Public Health and Community Medicine, Sahlgrenska Academy, University of Gothenburg, Sweden
[2]Angered Hospital, Hospitals West, Region Västra Götaland, Sweden
[3]Occupational and Environmental Medicine, Sahlgrenska University Hospital, Region Västra Götaland
[4]Department of Molecular and Clinical Medicine, Sahlgrenska Academy, University of Gothenburg, Sweden

Corresponding author: Mia Söderberg, Occupational and Environmental Medicine, Box 414, 40530 Gothenburg, Sweden.
Email: mia.soderberg@amm.gu.se

For peer review only

For peer review only - http://bmjopen.bmj.com/site/about/guidelines.xhtml

Abstract

Objectives: To investigate work and living conditions as determinants of COVID-19 infection risks in foreign-born workers in non-healthcare occupations.
Design: Data were collected according to a qualitative design, using semi-structured interviews. Verbatim transcripts of these interviews were analysed according to systematic text condensation.
Participants: We recruited foreign-born workers (n=15) and union representatives (n=6) among taxi drivers, bus and tram drivers, pizza bakers, cleaners, and property caretakers, all indicated as risk occupations during the COVID-19 in Sweden.
Results: Four overarching themes were found: "Virus exposure at work", "Aspects of low status and undervalued work", "Lack of access to information" and "Foreign-born persons' position". Virus exposure was frequent due to many social interactions over a workday, out of which several were physically close, sometimes to the point of touching. The respondents fulfilled important societal functions, but their work was undervalued due to low job status, and they had little influence on improving safety at work. Lack of health literacy limited foreign-born workers to access information about COVID-19 infection risks and protection, since most information from health organisations and employers was only available in Swedish and not adapted to their living conditions or disseminated through unknown channels. Instead, many turned to personal contacts or social media through which a lot of misinformation was spread. Foreign-born persons were also subjected to exploitation since a Swedish residency permit could depend on maintaining employment, making it almost impossible to make demands for improved safety at work.
Conclusions: Structural factors and lack of adapted information manifested themselves as fewer possibilities for protection against COVID-19. In a globalized world, new widespread diseases are likely to occur, and more knowledge is needed to protect all workers equally. Our results are transferable to similar contexts and bring forth aspects that can be tried in quantitative studies or public health interventions.

Strengths and limitations of this study

- This study recruited participants from an already vulnerable sociodemographic group that needed tailored public health interventions during the pandemic. However, since persons from this sub-population tend to not participate in scientific studies, little was known about their particular needs.
- The use of qualitative methodology increased the possibilities of exploring new themes and extracting in-depth information about such themes.
- The interviews were mostly conducted in the participant's native language, but some interviews were carried out in a language that was neither the respondent's nor the interviewer's native language, which likely restricted a deeper understanding.
- Those who volunteered may have consisted of a selection of more well-established persons in Swedish society. Moreover, almost all the participants were men.

For peer review only - http://bmjopen.bmj.com/site/about/guidelines.xhtml

Background

SARS-CoV-2 is an RNA virus that causes COVID-19, which had a pandemic spread over winter and spring 2020, reflected in high morbidity and mortality worldwide (1-3). When the World Health Organisation declared the outbreak as a pandemic (11th of March 2020), the Swedish Public Health Agency made formal requests to impose social restrictions, such as a maximum of people at social gatherings, that elderly people (>70 years) should avoid close social contacts, no outside visitors at elderly homes and that the teaching of upper secondary school pupils and university students should be done remotely (4). Otherwise, Swedish society remained open, with no curfews, and with shops, restaurants and elementary schools mostly operating as usual. In September 2020 when the prevalence of COVID cases was low most recommendations were relaxed, but re-introduced again in December 2020 when the numbers of cases increased rapidly. This meant stricter regulations for the allowed number of people in shops and social venues, earlier closing times for bars and restaurants, and recommendations to wear facemasks when travelling with public transport, although the latter was not mandatory.

In December 2020, the Swedish COVID-19 vaccination program started. The first batches were aimed at health and social care staff, the elderly, or those who were considered as risk groups due to a diagnosis such as cardiovascular diseases or diabetes. In June 2021, all adults ≥18 years were eligible for vaccination, which was expanded to everyone aged 16 years or older in July of the same year. The vaccination program was deemed successful as over 70% of the Swedish adult population has received at least one dose as of July 2021(4). As a result, almost all regulations were removed but were once again reinforced in December 2021 when the number of confirmed cases increased. Finally, most recommendations were removed in February 2022 and in March 2022 when COVID-19 no longer was considered a public and societal dangerous disease, the Swedish public health agency directed their main focus to increase vaccination rates and vaccination booster jabs (4).

Due to the absence of any treatment or vaccine during the first waves of the outbreak, research was foremost concentrated on clinical and pharmaceutical aspects. Yet, other public health concerns emerged as markers of a low socioeconomic position or belonging to an ethnic minority were associated with an increased risk of infection, severe illness, and death from COVID-19, increasing already existing health disparities between social groups (5, 6). Severe disease and death are associated with the prevalence of underlying diseases (7). Infection risks, however, is determined by the number of social contacts as COVID-19 is primarily transmitted through drops in sneezing, coughing, speech, or aerosol transmission, with the latter being exacerbated in crowded and poorly ventilated spaces. In people of working age, work is a dominant source of social contacts. However, there are markedly differences in possibilities to avoid virus exposure, as low-wage workers to a less extent can practice social distancing from the nature of their work tasks and to work from home, and reliance on public transport when lacking the economical means to own a car (8, 9).

When COVID-19 was declared a pandemic, work in healthcare and social care was promptly identified as a risk for becoming infected due to many close physical contacts. Yet, there were other occupations with many social contacts in which a high prevalence of COVID-19 infection was observed, and in which virus exposure and protection possibilities were less investigated. In a review study, including studies from the early stages of the pandemic, abattoir workers and related food workers, and retail staff displayed increased risks of contracting the coronavirus (10). Two Swedish register studies, investigating occupations outside the health and social care sector, showed the highest prevalence of a positive

For peer review only - http://bmjopen.bmj.com/site/about/guidelines.xhtml

Polymerase Chain Reaction (PCR) test for COVID-19 or death in COVID-19 (until May or June 2020) among taxi drivers, pizza bakers, and bus and tram drivers and a high prevalence among property caretakers and cleaners (11, 12). Another study, recording COVID-19 cases over the first 40 days since the first detected case in six Asian countries, noted the highest infection rates in taxi and van drivers and, in descending order, shop salespersons, religious professionals, and construction labourers (13).

As time progressed, similar and new at-risk occupations were found. A study from the UK with access to national register data until December 2020, showed the highest COVID-19 mortality rates in taxi drivers (Hazard ratios (HR) 2.01, 95% confidence intervals (CIs) 1.67–2.43, in men), followed by support staff, bus and coach drivers, sanitary workers and van drivers, adjusted for age, multiple sociodemographic factors and prior diagnoses (14). In a Norwegian national register study, the highest odds ratios (OR) for COVID-19 (a positive PCR test or an ICD-10 diagnostic code U07.1) over winter–spring 2020 was found in bus and tram drivers, taxi drivers, food counter attendants, bartenders, shops assistants and cleaners (15). During the winter of 2021, which constituted the second pandemic wave, public transport conductors, bartenders, travel stewards, waiters, food counter attendants, bus and tram drivers, taxi drivers, and hairdressers displayed the highest COVID infection rates (15). A report from the Swedish Social Insurance Agency, using sick leave data up until March 2021, illustrated a 42% increased relative risk (RR) of long-term sick leave (>21 days) in taxi drivers, compared to all other employed persons (16). Bus and tram drivers also displayed higher rates of both short-term sick leave (42% increased risk of at least one day of sick leave) and long-term sick leave (39% increased risk of >21 sick leave days).

Despite the observations of an increased risk of COVID-19 in several occupations with similar characteristics, little is known about infection risks in day-to-day situations as reported by the workers themselves. An interview study with fast-food workers in the US (n=15) provided some insight, such as customers refusing to wear facemasks or subjecting workers to verbal abuse or threats when they were being asked to practice social distancing or to wear a facemask (17). A British study based on interviews with bus drivers, delivery workers, and police staff, illustrated a lack of, or delayed, access to protective equipment and difficulties in distancing themselves from other people (18).

Higher risks of becoming infected may also interplay with having an immigrant background as most of the Swedish occupations with the highest occurrence of COVID-19 also employ the largest proportion of foreign-born workers: pizza bakers (79%), cleaners (57%), bus and tram drivers (48%) and taxi drivers (47%) (19). Analyses based on Norwegian national register data found that workers from Somalia, Pakistan, Iraq, Afghanistan, and Turkey displayed substantially higher ORs of a COVID-19 diagnosis than the general population, in particular bus- and tram drivers (OR 12.72, 95% CI 8.29-19.55), cleaners (OR 8.81, 95% CI 6.66-11.66) and taxi drivers (OR 6.56, 95% CI 4.88-8.86), age and sex-adjusted (20). Yet it was unclear if this was related to occupational risks or other factors, as foreign-born persons in the listed occupations mostly did not display higher ORs than other immigrants from the same country. Similarly, in a British registry study, the highest mortality from COVID-19 was found among taxi drivers, bus drivers, and cleaners, but the risk estimates decreased markedly (taxi drivers and cleaners) or were nullified (bus drivers) when adjusting for age, ethnicity, housing, living in a socio-economically vulnerable area and relevant diseases (21).

The reasons for increased infection risks in foreign-born workers are not clear. Besides virus exposure at work, one aspect that has been brought forth is the lack of access to crisis

For peer review only - http://bmjopen.bmj.com/site/about/guidelines.xhtml

information, as well as information on safety and rights at work (22, 23). Such barriers relate to "health literacy", a concept that can be briefly explained as the use of information to protect and improve health (24). From an individual perspective, the basic level involves having adequate language skills to consume and understand information correctly, while more complex features reflect the ability to acquire relevant information and how to apply it. Health literacy also reflects organisations' ability to adapt appropriate support for health-promoting behaviour. Another potential risk factor is poor housing conditions, which is more commonly found among foreign-born persons. Sweden displays some of the largest within-country gaps in inequalities in housing deprivation in Europe, as around 30% of all foreign-born persons live in overcrowded households, compared to 9% of Swedish-born persons (25). Overcrowding contributes to the spread of infection, and if co-habitants in such households work in risk occupations for COVID-19, it can exacerbate a circular infection pattern between the work and home environment.

In summary, increased COVID-19 infection rates have been found in many occupations outside the health and social care sector, and foreign-born workers in these jobs seem to be at particular risk. Based on existing evidence it can be assumed that increased infection relates to difficulties in social distancing, as well as barriers to accessing crisis information. Yet, exposure risks in the everyday situation as described by foreign-born workers themselves are largely unknown, partly because these occupations have received less attention but also since this sociodemographic group tend to be less prone to participate in scientific studies (26). This explorative study aims to increase the understanding of pathways for COVID-19 infection in foreign-born workers in non-healthcare occupations. We used a qualitative in-depth design to capture aspects that may not be adequately covered using questionnaires or other methods collecting quantitative data

Methods

Recruitment of participants

In order to complement prior studies on non-healthcare keyworkers and based on official statistics from the Public Health Agency of Sweden we chose to investigate the study objectives in pizza bakers, taxi drivers, bus and tram drivers, cleaners, and property caretakers (11, 12). The participants were recruited through convenience sampling, a non-random sampling method in which participants are invited through existing social networks. Initially, we only recruited foreign-born workers, but as the project progressed, we decided to include union representatives, who could contribute with an overview of the selected occupations in addition to their own experience of the working conditions. The union representatives did not have to be foreign-born. Two health guides from the East Gothenburg Health Centre were contracted for the recruitment: Juhaina Swaid (JS) (co-author) and Saido Mohamed (SM). In Sweden, health guides have a broad range of tasks such as facilitating communication between organisations and citizens, disseminating knowledge about how people can promote their health, and navigating the healthcare system to ensure that everyone in society has access to the same information. Through their work, JS and SM had a wide network of social contacts and an established trust with the people in their catchment areas. They could also conduct the interviews in several of the participants' native languages.

The participants in this study were recruited through 1) social contacts of the health guides JS and SM (n=13), and 2) social contacts of colleagues at the Department of Occupational and Environmental Medicine, Sahlgrenska University Hospital (n=2). The union representatives (n=6) were recruited by 3) emailing the unions' contact persons listed on the website for each

For peer review only - http://bmjopen.bmj.com/site/about/guidelines.xhtml

union. When contact was established, the person in question sent an invitation email to either a selection of union workers who might be interested in participating or to a global email list. In Sweden, most pizza bakers are self-employed or work in small family-owned businesses, and we were unable to find any union representatives for this occupation. The lack of union representatives for this restaurant workers' branch was confirmed by the Swedish Hotel and Restaurant Workers' Union.

Patient and public involvement
No patients were involved in this study.

Data collection
The workers were interviewed during March 2021–March 2022, and the union representatives were interviewed in February–March 2022. To encourage the participants to freely describe their situation the interviews were carried out according to an explorative and semi-structured methodology with sets of both open-ended questions and specific questions about the work environment. The interviews were carried out by first author Mia Söderberg (MS), and the two health guides JS and SM. MS had previous experience in collecting data according to qualitative methodology. JS and SM received supervision from MS on how to conduct the interviews. JS also participated as an observer during one of the interviews that MS conducted, but this was not possible for SM. Most of the interviews took place at the participant's workplace or by Zoom or telephone. A few of the interviews were carried out at the homes of the participants or a café in their neighbourhood. The reason for the different venues was to accommodate the wishes of the participants, out of which several were hesitant to participate, or worked long hours and had many responsibilities at home, and therefore requested to be interviewed by phone or Zoom, or near their homes in an environment where they felt comfortable.

The interview guide was formulated around of three main topics: 1) virus exposure and protective measures at work, 2) access to information, and 3) living conditions. Examples of the interview questions are: (virus exposure and protective measures) "How often do social contacts at work require other people to stand very close to you and shout to overcome the surrounding noise?", "What methods have you used to protect yourself from exposure to infection?", (access to information): "How do you obtain information about COVID-19?", (living conditions): "Are there any reasons why you would not call in sick if you had COVID-19 symptoms?". We also included an open question: "What do you think is the main reason for the spread of COVID-19?". The union representatives were asked the same questions both from the perspective of their own work conditions and the perspective of the members they represented. Both JS and SM had encountered some of the workers prior to this study through their work as health guides. For that reason, the interviewers took particular care to explain that the interviews and handling of any sensitive information were conducted according to professional secrecy. None of the other authors had prior contact with the participants. After the 15th interview with the workers, the content became repetitive and the data was perceived as saturated, therefore the recruitment ended. All union representatives who reported an interest in participating were interviewed.

Language considerations and transcription
The interviews were carried out by JS (native language Arabic), Saido Mohamed (SM) (native language Somali), and Mia Söderberg (MS) (native language Swedish). The languages of each interviewer are shown in Figure 1.

For peer review only - http://bmjopen.bmj.com/site/about/guidelines.xhtml

Figure 1. Languages used during the interviews.

None of the fifteen foreign-born workers had Swedish as their native language. Five of these interviews were conducted in Arabic by JS and four interviews in Somali by SM, i.e., an interview situation in the native language of both persons. The remaining six workers were interviewed in Swedish, of which four were conducted by JS, i.e., in a language that was neither the interviewer's nor the respondent's native language. With this in mind, JS revised the transcript of these interviews to evaluate if it corresponded with her perception of what emerged during the interviews and to clarify aspects that may not be apparent. The six union representatives were interviewed in Swedish by MS. Swedish was not the native language of three of the union representatives, but their language level was perceived as either equal to a native Swede (native language: Norwegian) or as an advanced Swedish speaker (native languages: Serbian and Persian). The interviews in Arabic and Somali were translated into Swedish by professional translators and all 21 interviews were then transcribed verbatim by the same company that translated the interviews. After this process, JS and SM contacted a few participants again to get further information on unclear content.

Analyses

The analysis of the data was primarily carried out by first author MS and second author Maria Magnusson (MM), who were both experienced in carrying out qualitative studies. MM has particular experience with systematic text condensation as proposed by Malterud (27), a descriptive and explorative method, which was the chosen analysis method of this study. This methodology is also characterised by paying particular attention to the participants' experiences and perspectives while emphasising an understanding of the underlying processes. MS and MM started by reading the entire transcript of all the interviews, without singling out meaningful information to get the overall gist of the material. Both authors then re-read the printed transcripts and marked all content that was perceived as meaning-bearing units with a focus on the study objectives. Other content was marked to be re-evaluated later.

In the next step, the marked units were revised and labelled into either relating to work, general living conditions, information access, or aspects of being foreign-born. From this step onwards, JS participated in the analysis process with interpretations and her professional and cultural knowledge. The themes were then further refined and presented for discussion among all the authors. The senior researchers, Kristina Jakobsson (KJ) and Annika Rosengren (AR) contributed with their expertise in public health and occupational research. The result was then edited and condensed into main themes with underlying sub-themes. The sub-themes were also evaluated with consideration to how often a certain theme was mentioned, or if the union representatives reported this as a common occurrence among the members they represented.

Results

Fifteen workers aged 30-50 were interviewed, and a majority were men (n=13). The workers were pizza bakers (n=4), property caretakers (n=2), taxi drivers (n=3), tram or bus drivers (n=3), and cleaners (n=3), out of which two worked as hospital cleaners. Time in their current occupation ranged from two to sixteen years. Six union representatives participated, three men and three women, representing property caretakers (n=1), cleaners (n=3), taxi drivers (n=1) and bus drivers (n=1). The union representatives worked with union assignments one to four days per week, the remaining time they worked in their main occupation. The number of unionised members they represented ranged from 300 to 7000 members. The countries of

For peer review only - http://bmjopen.bmj.com/site/about/guidelines.xhtml

origin of the participants were Turkey, Syria, Iraq, Palestine, Serbia, Somalia, Norway, Iran and Sweden. All the Swedish born participants were union representatives. Most of the participants lived together with their spouses and children. Further descriptive information about the study participants can be found in Appendix I.

Our analyses resulted in four overarching themes: 1. Virus exposure at work, 2. Aspects of low status and undervalued work 3. Lack of access to information and 4. Foreign-born persons' position. The main and sub-themes are presented in Table 1.

Table 1. Main and sub-themes

**Main Themes**	**Sub-themes**	
1. Virus exposure at work	1.1 Hard-to-avoid contamination risks	Social contacts<br>Surfaces
	1.2 Lack of safety culture	Employees<br>Employers<br>Employer liability
	1.3 Contributing to the spread of the disease	Social contacts over many venues<br>Social contact with vulnerable persons
2. Aspects of low status and undervalued work	2.1 Fulfilling an unappreciated societal role	Increased need for cleaning during the pandemic<br>Upholding the public transport system
	2.2 Occupational status influences workers' value and safety	Protective interventions deemed too expensive<br>Unequal protection
	2.3 Low authority over their work environment	Lack of influence<br>Distance to decision-makers
3. Lack of access to information	3.1 Inadequate health literacy	Workers<br>Employers<br>Organisations
	3.2 Consequences	Turned to unofficial information sources<br>Spread of misinformation
	3.3 Suggestions for improvements	Information in many languages<br>Utilizing complementary information outlets<br>Audio-based information
4. Foreign-born persons' position	4.1 Exploitation of a vulnerable Position	Need the job for a Swedish residency<br>Need to financially support others<br>Unawareness of regulations or rights
	4.2 Protective directives not adapted for persons outside the majority society	Cannot work from home<br>Cannot avoid public transport<br>Different social patterns

1. Virus exposure at work

For peer review only - http://bmjopen.bmj.com/site/about/guidelines.xhtml

Due to the nature of the work tasks and the organisational environment, the participants reported that it was nearly impossible to avoid being exposed to infection risks. Sub-themes for "Virus exposure at work" were sorted into "Hard-to-avoid contamination risks", "Lack of safety culture" and "Contributing to the spread of disease".

1.1 Hard-to-avoid infection risks.

The most notable risk for being exposed to infection appeared to be the many social contacts the participants encountered over a workday, out of which several were physically close, and could include touching other people. For example, some bus-, tram- or taxi companies did not re-install any protective screens between the driver area and passengers during the second wave (winter 2021) and demanded that passengers enter through the front doors to facilitate ticket inspection. The drivers estimated that they would then come in close contact with more than hundreds of people in a single workday. The bus- tram and taxi drivers also carried out mobility services for citizens with reduced functional ability, such as the elderly, physically disabled persons, or school children with special needs, who often needed hands-on support.

Bus driver: "*For me, the passenger is the cause of infection. I drive you to work, to your exercise, to the store, to the health centre. One guy who stood and talked to me last week* (comment: this interview was conducted in February 2021). *He was going to the health centre for a COVID test! What the h*ll are you standing here saying, I thought*".

Taxi driver: *"There are some who are ill. I help elderly people who can't sit down, put on their seat belts, or walk up the stairs: (Interviewer): Do you support them? (Taxi driver): Maybe I'll hold their hand, yes."*

Taxi driver: *"Many of them need help with the seat belt and maybe help with getting into the car…(if) they have a walker - you go out and meet them, if it's slippery outside, you have to hold their arm… then you open the door and you want to hold their head so that they don't hit their head on the ceiling… yes, we touch a lot of people, even if they can manage quite well on paper. But you can't let anything happen. Say we touch 50% of all adults. I would say 80% need help with the seatbelt."*

The participants also encountered many social interactions that were not part of their work tasks, foremost in crowded venues such as schools and shopping centres, and the hospital cleaners came into contact with patients and medical staff. Many cleaners also travelled by public transport between their daily assignments since they did not own a car, or to avoid parking fees and traffic jams, which exposed them to risks, especially since facemasks were not mandatory on public transport in Sweden. The bus and tram drivers would also use public transport to travel to the start and end stations of their shifts.

Hospital cleaner: *"The patients need help. We say no! This is not part of our job. But if they are old and can't find the buttons* (comment: the buttons used to call for help or to operate their bed), *then we will show them the buttons. Some of the patients are old, then you have to stand close and speak loudly."*

Hospital cleaner: *"Always when I enter this office, the doctor is sitting there. He doesn't want to leave. So, I wipe a bit there"* (comment: the participant cleans the area around where the doctor is sitting).

For peer review only - http://bmjopen.bmj.com/site/about/guidelines.xhtml

Union representative for cleaners: *"I have a car, but I don't want to drive. You end up in traffic jams and you can't find a parking space. There's a lot of time pressure in our work... Therefore, people are forced to go for example by subway, by bus. I can see that the people who use public transport do not use facemasks."*

The participants handled tools or surfaces that had been touched by many other people, especially the property caretakers and the cleaners who operated over large areas, cleaned many different surfaces, and handled waste. The hospital cleaners rarely had the time to sanitise their equipment despite serving hospital wards, waiting rooms, and toilets.

Hospital cleaner: *In our group, we are five staff, and we use the same materials every day. (Interviewer: How often is it cleaned?) We usually take care of our materials and stuff and machines once a week. (Interviewer: You all use the same materials and the same machines?) Yes. (Interviewer: The machines and tools you use, have you increased cleaning them?) No."*

1.2 Lack of safety culture

The workers made an effort to work safely and made many requests for protective equipment at the beginning of the pandemic, but also reported that this became less important as time passed, due to stress, feeling restrained by cumbersome protective equipment, or reduced fears of the disease.

Union representative for cleaners*: "In the beginning, it was a panic about protective equipment, that's when everyone wanted to have and have and have, and finally when we got enough cleaning materials and everything they asked for that's when people started to ignore it. People eventually got used to it and said, "But nothing happens."*

Some employers introduced safety procedures and provided their workers with personal protective equipment in the early phases of the pandemic, while others seemingly delayed the provision of protective equipment and only after being pressured. The participants speculated that this was related to employers' lack of knowledge of best practices, not enough resources, or that they were unmotivated to protect their staff in a "soon-to-be-over" crisis.

Hospital cleaner: *"We got facemasks recently. The last time when corona was getting worse... they got new rules for us that we have to use facemasks* (comment: they received facemasks in October 2020)*."*

Bus driver: "*No, we didn't receive any recommendation from April to May 2020. No one warned us about this. It came after the number of infected people increased. Then the company and the municipalities began to take action and give recommendations. They started with it after the month of July* (comment: July 2020)."

Hospital cleaner: *"In the beginning, they sent us to a department called ICU and it was full of patients who had had COVID. We went there without protection, without mouth protection"* (comment: the unit was cleared of patients before cleaning). *There are so many instructions, and we know all the instructions but in the workplace, it is the opposite. It is the opposite."*

Hospital cleaner: *The emergency department built a barrack for examinations. Those who came were unwell. They had Corona or fever, or something. We asked why we are cleaning there - it's a risk! He* (comment: their manager) *said you must do it, there is no risk."*

For peer review only - http://bmjopen.bmj.com/site/about/guidelines.xhtml

There was also a feeling that employers only made changes that looked good “on paper” but neither cared for and sometimes even contradicted safety protocols. For example, managers ignored when staff came to work with obvious COVID-19 symptoms or that lunch breaks were scheduled so that far more than eight people would be gathered in a small lunchroom that was not properly cleaned or sanitized. People who spoke up could be punished by being assigned “the graveyard shifts” or not receiving enough work to fill a full-time schedule.

Hospital cleaner: *“They say we can't have staff meetings because of being too many. When we’re in the lunchroom there are more than eight people there... But I have to sit and eat!... The boss, we think he should make (changes). He doesn't't!* (Comment: Eight people was the maximum allowed number of people at social gatherings at the time of the interview)”.

Unclear employer liability also made it difficult to demand safer working conditions. For example, mobility services carried out by taxi drivers were often purchased by the local borough, while one company owned the vehicle, and another company might employ the driver. There was also uncertainty regarding who was responsible for the protection of cleaners against social contacts at shopping centres or in schools.

Union representative for bus drivers: “*There are several responsible persons… the one who owns the car, the company we work for, and then the one who procured the travel service - therefore there is no one to report problems to… I got frustrated and emailed the travel services committee* (comment: a committee of local politicians)*, one, two, three different persons, and got no answers*”.

<u>*1.3 Contributing to the spread of the disease*</u>

Some participants worried that their work contributed to the spread of COVID-19 to other, sometimes vulnerable, people. When conducting mobility services, the taxi drivers drove up to four passengers who were sometimes picked up from crowded venues, such as community centres or schools. This caused concern as many passengers could be considered risk groups for severe COVID-19 due to old age or poor health. The hospital cleaners met both healthcare staff and patients, which meant there was a large risk of contracting and spreading the virus.

Union representative for taxi drivers: *“This is quite a large day centre… say there are 20 people, plus regular staff… and there are maybe 5-6 other residents who go to other daily activities and there are staff so if you think of the infection... I pick up this girl and then I have to pick up a regular service traveller and there is an elderly man with a facemask, and I have to put him in the back seat with her* (comment: the girl that was picked up from the service centre) *and that didn't feel good. (Interviewer: Did she have a facemask?) No no, no possibility of wearing a facemask no... it was so offensive against everyone involved.”*

2) Aspects of low status and undervalued work

Despite providing important societal services there was a feeling that because they worked in what was considered low-status job, their safety and the work they performed were not considered important and valued. These aspects were sub-categorised into “Fulfilling an unappreciated societal role”, “Occupational status influences workers’ value and safety” and “Low authority over their work environment”.

<u>*2.1 Fulfilling an unappreciated societal role*</u>

Many participants had expanded responsibilities during the pandemic and were proud of upholding important functions and contributing to the battle against COVID-19. They received appreciation for their hard work but worried that this would be forgotten once the pandemic was over. There was also anger that contractors demanded extra work but did not want to offer any additional pay.

Union representative for bus drivers: *"We feel very forgotten in this. If we stop all traffic, then half of Sweden stops because then the health care workers can't reach their destination, then the teachers and students can't reach their destination. We are equal, we are not health care staff, but we are important in society."*

Union representative for cleaners: *"I think we are an important professional class, but in society, it is classified as a low professional class… We got a lot of extra orders, and they were very positive, and they were grateful for the cleaning and thanked all the cleaning companies in the community. But guaranteed, they will forget everything when the pandemic is over, unfortunately."*

2.2 Occupational status influences workers' value and safety

Many companies seemed to have treated the workers' safety as a balancing act between protecting their staff and a loss of revenue. Some transport companies did not want to install protective screens or restrict the number of allowed passengers per taxi car during the second wave (winter 2021), since that would require extra cars and staff. There were also bus companies that resisted introducing facemasks to not "scare the passengers". When facemasks or visors became mandatory this posed new problems as wearing a visor would fog-up glasses, or could cause mirror reflections, endangering driving. Some buses received a driving ban due to these mirror reflections but were put into service anyway. The union demanded restrictions from passengers entering the area near the driver, but this was met by resistance since it would make ticket inspections impossible, leading to losses in revenue.

Union representative for cleaners: *"When the first wave came, I contacted some employers to make checklists with them regarding infection (risks), they would then say, "But they already have gloves!"*

Union representative for bus drivers: "*They lost 1.6 billion (Swedish krona) last year. If you divide 1.6 billion by the number of drivers, you know the price of my life. Someone who sits in an office decides they want to gain money in this way* (comment: a loss equal to around USD 160,400,000)*."*

Union representative for taxi drivers: *"Then the infection increased and it's like, well… the front seat was closed again at the beginning of January. So, during this whole giant wave, it was open… it was awful… I called my boss and said, "I won't put anyone in the front seat". He completely panicked and said, "You can't do that", "well yes actually I can because it's about my life and health* (comment: confirmed COVID infection cases increased rapidly in November 2021, and the front seat was closed for passengers January 2022)*".*

The hospital cleaners were initially refused antigen tests at work, despite that this was freely available for the healthcare staff working on the same premises. Instead, the cleaners had to go to their local healthcare centre to get tested and were not allowed to work while waiting for the results, which meant a loss of income.

For peer review only - http://bmjopen.bmj.com/site/about/guidelines.xhtml

Hospital cleaner: *There is a special test at the hospital for staff there. But they sent us to our health care centre… We have to make an appointment ourselves and stay at home for four days because of this until we get an answer. Now I think it will be better… but this thing started two months ago* (comment: free antigen tests were available to the cleaners at their workplace in February 2021).*"*

<u>*2.3 Low authority over their work environment*</u>
Most of the workers had limited possibilities to influence their work conditions or to make their demands and concerns heard by the management or the employers. The people who made the decisions on safety rules and other work-related aspects were far removed from the day-to-day work and had limited knowledge of the actual work content. There was also anger that those in charge worked in a safe environment from home, did not listen to workers or union representatives, and seemingly looked down on the workers.

Union representative for taxi drivers: *It's about us being just taxi drivers. Yes, and that's the hierarchy, yes… and it's really sad to say it, but that mentality is out there* (comment: among the decision makers)*, "they are dumb taxi drivers", it's impossible to get away from that."*

Union representative for bus drivers: "Y*ou sit at home, in your safe quiet living room while I'm out there driving among people and (you) talk about how I should feel at my workplace?! Damn it! Do not sit at home in your living room and think you know better than me about my workday."*

3) Lack of access to information
Throughout the interviews, it was clear that faltering health literacy resulted in a lack of access to vital information regarding infection risks and protective measures. This theme was sorted into the sub-themes "Inadequate health literacy", "Consequences" and "Suggestions for improvements".

<u>*3.1 Inadequate health literacy*</u>
Almost all participants reported that they, their colleagues, or some of the union-affiliated members they represented had insufficient language skills to properly understand crisis information in Swedish, especially if such information was formulated in a bureaucratic style, or with ambiguous phrasings. Due to a lack of information about rights even before the pandemic, many were also unsure what demand they could make regarding safety at work and provision of protective equipment when COVID-19 spread through society.

Taxi driver: *"The news reporting about the disease was in Swedish, there was no Arabic channel that gave information about it in Arabic or other languages. There are many people in Sweden who do not know Swedish. They know limited Swedish, which is not good enough."*

Pizza baker: "*What I don't understand is – there's no requirement for facemasks. And then they say that we should wear facemasks when it is… high time? Or when it is rush hour? I cannot know what time it is rush hour… should I wear a facemask or not?*"

Union representative for cleaners: *"As I said, we have a bit of a problem with the language. People might feel worried if they want to complain or report something, they don't know that*

For peer review only - http://bmjopen.bmj.com/site/about/guidelines.xhtml

they have the right to do so. They get scared and don't dare say anything. That's the lack we actually have."

Health literacy is a two-sided process and involves the competency of the information disseminator, but as of winter 2022, many employers still only distribute safety information in Swedish and through ineffective methods. Taxi, bus, and tram drivers and property caretakers, whose routes and assignments need to be coordinated, received information via staff apps or web platforms. For most others, information was spread through methods such as text messages or leaflets on the staff noticeboards. This information was mostly in Swedish, and the participants figured that their employers knew that some workers wouldn't understand, but that it "looked good on paper". Some participants received oral information at staff meetings, but this was also in Swedish.

Union representative for cleaners: *"They have an information board in the office, people go by and collect materials or gather there. But they just put information on the board, I think you should say that this information applies to Corona so go and read it for example. (Interviewer: They think they have done their employer responsibility, but they don't check that people actually read and if they understand?) No exactly."*

Bus driver*: "The Swedes are a people who trust the government, (but) the Swedish media have not explained clearly, in a good way about corona for migrants or new arrivals, the consequence of this was that a large part of them died from corona."*

Hospital cleaner: *"In our job, there are many foreigners. They don't understand the language very well. Sometimes they sit and listen* (comment: during the staff meetings) *but they don't understand anything."*

<u>*3.2 Consequences*</u>

The lack of adapted information from employers and official outlets resulted in almost none of the workers using official public health institutions as a source of information, at least not during the first stages of the pandemic. For many of them the Public Health Agency of Sweden or the health care information organization "1177", the main distributors of COVID-related information, was unknown. The workers primarily received their information from news channels in their country of origin, from friends and relatives, or social media, through which a lot of misinformation was spread. Some participants also mentioned institutions that they were familiar with and trusted, such as their local healthcare centre or pharmacist, as reliable information sources.

Taxi driver: *"At the beginning, a friend, a doctor, said that corona did not exist. Such information has confused us a bit, hasn't it? A doctor… who says that there is no such thing as corona. In the beginning, I heard many rumours, that there are many old people in Sweden that they want to get rid of, right? In the beginning, we heard a lot of contradictory information."*

Property caretaker: "*(Interviewer: 1177 have a webpage that is translated into Arabic. If something happens, would you call…?). I did not make contact with anyone because of what was spread in the media, Facebook, and from the doctors. It was different (conflicting) information. I thought it was not beneficial. (Interviewer: If you want to obtain information about COVID-19, which information source do you trust?) My relatives."*

For peer review only - http://bmjopen.bmj.com/site/about/guidelines.xhtml

Taxi driver: *"(Interviewer: Which source of information do you trust the most?) Ah…TV. There are many people who watch TV, and the radio as well.* (Comment: TV and radio from their home countries)*."*

Property caretaker: *"(Interviewer: Do you trust the authorities?) No, I don't. I am newly arrived in Sweden, there are many websites that are linked to the authorities that I am not aware of. I don't know who to contact. I might contact the health centre or the pharmacy."*

3.3 Suggestions for improvements

The respondents made several suggestions on how information dissemination could be improved, primarily emphasizing the need for information in more languages than Swedish. During times of less strict regulations, information could be disseminated at outdoor meetings. Some participants also expressed a sense of trust in health centres or pharmacies, and that these health institutions could have an expanded role in providing the local community with information. There were also suggestions that information regarding responsibilities and how to conduct safety at work could be sent by post to all people who were registered as running businesses. Since some immigrants, especially older people, have difficulties reading and writing, some also thought it would be beneficial to produce audio-based information, which could be disseminated on government websites.

Taxi driver: "My grandfather will not understand printed information if you give him this leaflet, information should be in audio format and easy to understand."

Property caretaker: *"Authorities who know that I run a business should have contacted me, they should have sent me a letter or texted me."*

4) Foreign-born persons' position

Some aspects were primarily tied to having an immigrant background and these were sorted into the sub-themes: "Exploitation of a vulnerable position" and "Protective directives not adapted for persons outside the majority".

4.1 Exploitation of a vulnerable position

One of the most salient observations from the union representatives was difficulties in improving protection in cases where employment was a prerequisite to upholding a Swedish residency permit. As told by some of the union representatives, there are several Swedish vocational training programs designed for new immigrants and therefore a high proportion of foreign-born persons without permanent residency in some occupations, which sometimes could be taken advantage of. Some employers also appeared to be aware that many immigrants need to financially support their relatives, which also might be abused, as speaking up against poor working conditions could result in fewer working hours or being assigned the worst shifts. Such aspects were also a reason for not joining the union since the union does not accept unregulated overtime.

Union representative for bus drivers: "*We often notice that newly arrived persons attend SFI* (comment: Swedish For Immigrants) *training programs and the vocational bus training program. You are then completely dependent on your boss to get a residency permit. They would rather drive and get the wrong salary to get a residence permit. Because of this, I have chosen representatives against these employers who actually choose to exploit these people.*

For peer review only - http://bmjopen.bmj.com/site/about/guidelines.xhtml

This is almost modern slavery, "you do as I say, or you go home" (comment: she takes extra care to choose union representatives to combat these issues)".

Union representative taxi driver: *Many of them then want to work a lot, they want money. Many send money home, many come from vulnerable (residential) areas so their children should get the same things as other children therefore they are at a disadvantage. Our driving and rest time rules mean that they are quite limited in how much they can work. If you're in the union, you can't cheat. (Interviewer: Do the employers know about this?) Oh yes. (Interviewer: Do you think this is the case in your industry?) Yes absolutely."*

4.2 Protective directives not adapted for persons outside the majority society

The participants reported that many of the safety recommendations disseminated by the Public Health Agency of Sweden, such as working from home or avoiding public transport, could not be adapted to the participants' life situation. One new theme that arose, for which there was no adapted information, was large social gatherings with persons from the same country, often privately arranged at cafés or other local venues. Other examples were large gatherings in cramped local food stores, mostly owned by other immigrants.

Pizza baker: *"We have a cafe where you gather after work… How many were there last Friday or Saturday…I don't think there were more than 40 people.* (Comment: the interview was conducted when eight persons were the maximum number of allowed people at social gatherings).*"*

Pizza baker: *"The (local) grocery store, that's it, 1000 people who come to the grocery store, move all the time, (touch) all the stuff, the bags, so there's a risk in the grocery store."*

One aspect of interest in this study was overcrowding, but none of the interviewed persons could be defined as living in overcrowded conditions. Some of the union representatives mentioned that their members lived in overcrowded housing, but since none of the respondents themselves lived like this we could not ask for more detailed information. Only two workers and one union representative reported limited finances as an obstacle for sick leave. Otherwise, most of the respondents reasoned that one's health was more important than money or that they were financially secure enough to go on sick leave if they needed it.

Discussion

This explorative interview study resulted in four overarching themes. The first concerned virus exposure at work, mainly through large numbers of social interactions and a lack of safety culture. The second theme related to working in a society-deemed low-status occupation where the participants' work and they as workers were not adequately appreciated or protected, despite carrying out important tasks for societal function. The third theme revolved around a lack of health literacy which meant that participants had limited access to essential crisis information, and instead turned to other information channels, through which a lot of misinformation was spread, causing confusion and the use of maladapted protective behaviour. Lastly, there were risk aspects tied to being foreign-born, most notably a precarious position in the labour market and the need to keep an employment to maintain residency in Sweden, which could lead to exploitation and difficulties in speaking up against hazardous working conditions. There was also a lack of directives that were adapted to persons outside the majority society.

For peer review only - http://bmjopen.bmj.com/site/about/guidelines.xhtml

Virus exposure at work

Concerning virus exposure at work, the media and public health organisations have mainly focused on the health and social care sector, but other industries and large occupational groups also experienced increased risks of virus exposure at work (9-12). International studies have consistently found that working in a low-status occupation is associated with an increased risk of COVID-19. In this study, we took a closer look at five Swedish occupational groups (11, 12). Weighing in hard-to-avoid sources of potential infection and poor organisational safety culture, cleaners, taxi, bus, and tram drivers seemed notably vulnerable to many and physically close social contacts, which may explain why infection and mortality remained high among bus, tram, and taxi drivers throughout the pandemic (15, 16). The many social contacts could also contribute to an increased spread of the disease to other people, some of whom could be considered risk groups for developing severe COVID-19.

A few studies with taxi- and public transport drivers have presented similar findings (28), while little is known about cleaners, property caretakers, and pizza bakers, with the latter perhaps being best comparable to fast-food workers from an international perspective. An American study among fast-food workers with data collected from both questionnaires (n=417) and interviews (n=15) (18) illustrated several occupational hazards. These were partly linked to employers' inadequate investment and interest in safety culture, such as not providing staff with facemasks. Forty percent of workers needed to buy their own facemasks, and out of those, around 10% of respondents were unable to afford them. Many co-workers or customers did not wear facemasks, with frequent incidents of threats and verbal abuse from customers when the staff tried to make them adhere to safety protocols (17). In contrast, the pizza bakers in our study were mostly self-employed and appeared to be very able to create protective routines that were respected by staff and customers.

Aspects of low status and undervalued work

Amid the pandemic, the inequities between different groups of workers became apparent, as workers in higher-status jobs could protect themselves, for example by working from home, while those in already precarious jobs needed to work on-site, exposing themselves to the risk of infection. In most countries, including Sweden, healthcare workers were hailed as heroes during the pandemic. However, the workers in our study, who also provide important functions for the upholding of society, did not receive the same widespread acclaim. Bus, tram, and taxi drivers sustained the transport infrastructure and cleaners had an enhanced role since increased cleaning was regarded as an important measure to prevent the spread of COVID-19. One qualitative study from the UK (18), conducted among bus drivers and transport workers, found similar themes as this study, primarily a sense of being unprotected at work, late or no introduction of protective equipment, as well as comparing themselves with other essential workers but without the same level of appreciation.

The definition of a low-status job is often related to a lack of longer formal training requirements or how work tasks are valued. Our investigated occupations can, by this definition, be viewed as low-status jobs. As such, one respondent reported that the employer calculated the financial loss for protective interventions and weighted this against the safety of their staff. The distance between the workers, decision-makers, and employers also resulted in frustration since the people in authority often lacked knowledge of their work conditions and how to create a protective work environment.

Lack of access to information

For peer review only - http://bmjopen.bmj.com/site/about/guidelines.xhtml

Another finding was the double-sided lack of health literacy from both the receiver's and the sender’s perspectives. When COVID-19 was declared a full-scale pandemic, it dominated the news flow worldwide and awareness of the disease was high, but crucial information on what constituted a risk for infection and protective measures, did not reach everyone equally. An EU-funded consortium (COVINFORM) concluded that morbidity in COVID-19 partly depended on “Vulnerability in relation to communication”, meaning lack of access to, or poorly adapted, information (29). A Swedish study conducted before the pandemic illustrated that in geographical areas marked by low socioeconomic status and with a large proportion of first- and second-generation immigrants, there is a higher use of social media, foreign news media, or social networks as the main source of information, especially among people with limited Swedish language proficiency (30). This difficulty in accessing important societal information is referred to as “the knowledge gap”, and has been observed in several countries, in which foreign-born persons have more difficulties accessing crisis information since they lack social connections with knowledge about the new country or that they distrust authorities (23). Health literacy tends to focus on the skills of the recipient, but it is indeed a two-way process in which the disseminator, in this context, employers and the Swedish health authorities, have a responsibility for disseminating information in a manner that is both received, understood and adapted to the communication patterns of the receiver. This is not only the ethically correct approach but also has a bearing on fundamental human rights in being given the possibility to protect one’s health.

Foreign-born persons’ position

International studies have nearly unanimously concluded that the risk of contracting COVID-19 is higher in immigrants in low-status jobs, a group of workers that even before the pandemic were at larger risk of work-related ill-health. A review study by the Swedish Work Environment Authority showed that individuals with an immigrant background more often worked in stressful jobs that required short formal training and in these occupations were assigned the worst tasks or received the least safety training (31). Unfortunately, people from these social groups tend to not participate in scientific studies, partly due to a lack of trust, but also since participation often requires language proficiency to, for example, fill in questionnaires. Studies based on self-reported data are then not only scarce but also often biased, reflecting the experiences of those who are more integrated into Swedish society.

One of the most alarming findings in this study was the occurrence of exploitation, as some employers appeared to abuse the fact that many immigrants are dependent on their employment to keep their Swedish residency permit. These findings correspond with studies from other countries in which immigrants, especially those newly arrived, more often work in precarious work situations due to problems entering the labour market and the dependency on their jobs to legally remain in the new country (32, 33). Furthermore, most of the occupations investigated in this study require short or no formal training and no need for high language skills, which likely means that there is a large pool of available workers and that it is easy to replace people who are perceived as difficult or demanding. Several union representatives reported that speaking up could lead to punishments such as being assigned the “graveyard shifts” or getting too few hours to fill a work week. One qualitative interview study among foreign-born cleaners in Sweden (34) illustrated a high competition between cleaning companies, but also many available workers which contributed to difficulties in receiving enough shifts to fulfil 100% employment. Due to these factors, the best shifts and a full-time schedule were often given to the workers who did not complain and who were on good terms with the foremen and employers. This made it difficult, if not impossible, to demand

For peer review only - http://bmjopen.bmj.com/site/about/guidelines.xhtml

improved work conditions. If similar tendencies exist in the occupations selected for our study, it further illustrates how the workers are exposed to large risks at work, as well as being powerless over the conditions that expose them to a potentially lethal disease. Little is still known about long-term ill-health from COVID-19, but this already vulnerable group may also face higher risks of future reduced workability from post-COVID (35). In light of such disparities, there has been a debate as to whether health organisations such as the WHO have a responsibility to enforce directives to protect workers (36).

Other considerations

This study had an explorative approach and used a set of questions with the possibility of follow-up questions to investigate the study's aims. Because of the qualitative design, external validity and generalizability cannot be tested. The participants commonly described similar occurrences of risk situations for exposure to the SARS-CoV-2 virus, and the stability of these responses indicates that the results have transferability to similar job situations. This was further reemphasized by the interviews with union representatives that have an overview of each occupation from representing large numbers of unionized members.

Our findings concur with issues of lack of information to all members of society, brought forth by the COVINFORM which overviews all the EU member states (29). One of the consortium's members, the Birmingham Local Outbreak Engagement Board, have introduced several policies and trials of accessible COVID-19 information to the city of Birmingham (UK) home to 187 different nationalities (37). Foremost, establishing multiple information channels, together with local organizations, community centres, faith settings, schools and faith institutions. This stands in contrast to a similar try-out in Norway, aimed at the Somali community, which was less successful due to not using already established communication channels (38). As many shortcomings in the Swedish strategies can be traced to a communication distance between policy makers and some groups in societies, future policies should aim at giving people outside the majority society the opportunity for influence, by being provided with communication channels to those responsible for compiling and disseminating information. In this way, knowledge of communication channels, special needs, and unknown risks can increase, and public health interventions can be designed with better precision.

Finally, there is a risk that a study on this particular topic may support a discourse of blame and stigmatisation. Even before the COVID-19 pandemic, the public debate concerning certain demographic groups and disadvantaged geographical areas was characterised by a negative and one-sided approach. Such problematic images are at risk of being exacerbated, as certain urban areas or ethnic groups displayed a higher occurrence of COVID-19, at the expense of understanding the underlying causes. It is therefore important that future studies acknowledge the existing structural inequalities to avoid that groups with limited resources to govern their life situation being blamed for increasing the spread of a disease.

Limitations

Based on the language proficiency of the interviewers and the respondents, we only conducted interviews in Swedish, Arabic, and Somali. Four interviews were conducted in a language (Swedish) that was neither the respondent's nor the interviewer's native language, which likely restricted a mutual deeper understanding. This was possibly restricted further by that some interviews were conducted by telephone or zoom, creating a larger communication distance. It is also likely that those who volunteered were a selection of more well-established people and with a higher level of trust in Swedish society and that we failed to recruit those

For peer review only - http://bmjopen.bmj.com/site/about/guidelines.xhtml

most at risk. Future studies should conduct data collection in every participant's native language and preferably by someone who also is knowledgeable in the participants' culture. There should also be more efforts to facilitate trust so that people who can be defined as marginalized feel comfortable to participate.

Almost all the participants were men since most of the selected occupations are male dominated. Nevertheless, foreign-born women more often report poorer working conditions or leaving the labour market prematurely, compared to both foreign-born men and Swedish-born women (27). Because of the male majority, aspects of dual vulnerability based on gender and immigrant status could not be elaborated. The three cleaners all worked at hospitals, while the union representatives all worked as general cleaners. Also, since none of the participants could be considered as living in an overcrowded household, we could not investigate this aspect.

Strengths

This study has several strengths. Foremost, that we could recruit participants from sociodemographic groups that previously have been identified as most at-risk of COVID-19 infection, but who usually do not participate in scientific studies (22). The lack of knowledge of the circumstances in such groups leads to a risk that public health interventions are not designed for those who need them the most. The successful recruitment was made possible because of the contracted health guides who, through their work, had a broad trust-based social network and were able to conduct several of the interviews in the participant's native language. Since relatively little is known about the work hazards and exposure risks of these groups in their private life, it is also an advantage that we have used qualitative methodology, which is an effective tool to explore new perspectives, since it makes it possible to capture nuances and delve into new themes with follow-up questions. We also interviewed both workers and union representatives. Through the latter, we could receive a broader perspective and gain information on negotiations with employers. Additionally, all union representatives were interviewed in February and March 2021, which meant they could contribute with knowledge from different phases of the pandemic.

Conclusions

In an increasingly globalised world, pandemics are likely to reappear and when widespread diseases enter all members of society should be protected equally. This interview study highlights several potential risks for exposure to the COVID-19 virus and the lack of protection possibilities in jobs outside the health and medical care sector dominated by foreign-born workers, as well as problems with the lack of adapted information. Our results are transferable to similar contexts and bring forth aspects that can be further explored in quantitative studies and public health interventions.

Funding

This study was funded by the Swedish Research Council for Health, Working Life and welfare (grant number: 2021-00326) and by the Swedish Research Council (grant numbers: 2021-06525).

Competing interests

None

For peer review only - http://bmjopen.bmj.com/site/about/guidelines.xhtml

Data availability statement
Data are not publicly available. Anonymized data are available upon reasonable request from the first author and pending fulfilment of legal requirements.

Ethics statements
Patient consent for publication. This study was approved by the Swedish Ethics Review Authority (2021-00177).

Author contributions
MS, MM, KJ, and AR conceived the study and were part of the overall conception and design of the study. MS coordinated the data collection and drafted the manuscript. MM and JS contributed to the data collection. All authors participated in the analysis of the data and critical revision of the manuscript. All authors have read and approved the final manuscript.

References
1. Cheng VC, Lau SK, Woo PC, Yuen KY. Severe acute respiratory syndrome coronavirus as an agent of emerging and reemerging infection. Clinical microbiology reviews. 2007;20(4):660-94.
2. Wu P, Hao X, Lau EH, Wong JY, Leung KS, Wu JT, et al. Real-time tentative assessment of the epidemiological characteristics of novel coronavirus infections in Wuhan, China, as of 22 January 2020. J Eurosurveillance 2020;25(3):2000044.
3. Burn-Murdoch J, Romei V, Giles C. Global coronavirus death toll could be 60% higher than reported. Financial Times. 2020.
4. The Public Health Agency of Sweden. When did what happen during the pandemic? Available from: https://www.folkhalsomyndigheten.se/smittskydd-beredskap/utbrott/aktuella-utbrott/covid-19/folkhalsomyndighetens-roll-under-arbetet-med-covid-19/nar-hande-vad-under-pandemin
5. Khanijahani A, Iezadi S, Gholipour K, Azami-Aghdash S, Naghibi D. A systematic review of racial/ethnic and socioeconomic disparities in COVID-19. International journal for equity in health. 2021;20(1):1-30.
6. Magesh S, John D, Li WT, Li Y, Mattingly-App A, Jain S, et al. Disparities in COVID-19 outcomes by race, ethnicity, and socioeconomic status: a systematic review and meta-analysis. AMA network open. 2021;4(11).
7. Schultz WM, Kelli HM, Lisko JC, Varghese T, Shen J, Sandesara P, et al. Socioeconomic status and cardiovascular outcomes: challenges and interventions. Circulation. 2018;137(20):2166-78.
8. Marmot M, Allen J. COVID-19: exposing and amplifying inequalities. Journal of Epidemiology and Community Health. 2020;74(9):681-2.
9. Baker MG. Who cannot work from home? Characterizing occupations facing increased risk during the COVID-19 pandemic using 2018 BLS data. Journal of Medrxiv. 2020.
10. Acke S, Couvreur S, Bramer WM, Schmickler M-N, De Schryver A, Haagsma J, et al. Global infectious disease risks associated with occupational exposure among non-healthcare workers: a systematic review of the literature. Occupational and Environmental Medicine, 2022;79(1):63-71.

For peer review only - http://bmjopen.bmj.com/site/about/guidelines.xhtml

11. Billingsley S, Brandén M, Aradhya S, Drefahl S, Mussino E, Andersson G. Deaths in the frontline: Occupation-specific COVID-19 mortality risks in Sweden. Report from University of Stockholm; 2020:36.
12. Public Health Agency of Sweden. Förekomst av covid-19 i olika yrkesgrupper. Bekräftade covid-19 fall i Sverige 13 mars-27 maj 2020. (Translated: The occurrence of COVID-19 in different occupational groups. Confirmed COVID-19 cases in Sweden 13 March-27 May 2020.) Östersund; 2020.
13. Lan F-Y, Wei C-F, Hsu Y-T, Christiani DC, Kales SN. Work-related COVID-19 transmission in six Asian countries/areas: A follow-up study. PlosOne. 2020;15(5).
14. Nafilyan V, Pawelek P, Ayoubkhani D, Rhodes S, Pembrey L, Matz M, et al. Occupation and COVID-19 mortality in England: a national linked data study of 14.3 million adults. Occupational and Environmental Medicine. 2022;79(7): 433-41.
15. Magnusson K, Nygård K, Methi F, Vold L, Telle K. Occupational risk of COVID-19 in the first versus second epidemic wave in Norway. Journal of Eurosurveillance. 2021; 26(40).
16. Effekter som covid-19 har på sjukförsäkringen. (Translated: Effects of COVID-19 on health insurance). The Swedish Social Insurance Agency; 2022.
17. Justie B, Koonse T, Macias M, Ray J, Waheed S. Fast-Food Frontline: COVID-19 and Working Conditions in Los Angeles. University California Los Angeles (UCLA); 2022.
18. May T, Aughterson H, Fancourt D, Burton A. 'Stressed, uncomfortable, vulnerable, neglected': a qualitative study of the psychological and social impact of the COVID-19 pandemic on UK frontline keyworkers. BMJ Open. 2021;11(11).
19. Statistics Sweden (Statistiska Centralbyrån). Yrkesregistret med yrkesstatistik 2017. Yrkesstrukturen i Sverige. (Translated: The occupational register with occupational statistics 2017. The occupational structure in Sweden.) Stockholm: Statistics Sweden; 2019.
20. Kjollesdal MK, Magnusson KJM. Occupational risk of COVID-19 by country of birth. A register-based study. Journal of Public Health, 2021: 1-7.
21. Mutambudzi M, Niedzwiedz CL, Macdonald EB, Leyland AH, Mair FS, Anderson JJ, et al. Occupation and risk of COVID-19: prospective cohort study of 120,621 UK Biobank participants. Journal of MedRxiv. 2020.
22. Ahonen EQ, Benavides FG, Benach J. Immigrant populations, work and health—a systematic literature review. Scandinavian Journal of Work, Environment & Health. 2007:96-104.
23. Spence PR, Lachlan KA, Burke JA. Differences in crisis knowledge across age, race, and socioeconomic status during Hurricane Ike: A field test and extension of the knowledge gap hypothesis. Journal of Communication Theory. 2011;21(3):261-78.
24. Berkman ND, Sheridan SL, Donahue KE, Halpern DJ, Crotty K. Low health literacy and health outcomes: an updated systematic review. Annals of Internal Medicine. 2011; 155(2): 97-107.
25. Ayala L, Bárcena-Martín E, Cantó O, Navarro C. Working Paper No. 73: 2021. Housing Inequalities in COVID-19 Lockdown Across European Countries. Vigo, Spain: EQUALITAS.
26. Magnusson M: Bortfallet och folkhälsan. Conference report ”Varför svarar de inte på våra frågor?” (Translated: "Why don't they answer our questions?"). Socialmedicinsk tidskrift. 2015, 92(1):89-96.
27. Malterud K. Systematic text condensation: a strategy for qualitative analysis. Scandinavian Journal of Public Health. 2012;40(8):795-805.

For peer review only - http://bmjopen.bmj.com/site/about/guidelines.xhtml

28. James K, Thompson C, Chin-Bailey C, Davis KD, Nevins DH, Walters D, et al. COVID-19 related risk perception among taxi operators in Kingston and St. Andrew, Jamaica. Journal of transport. 2021;22.
29. Molenaar J. The COVINFORM project - Migrants as a 'vulnerable group' in the COVID-19 pandemic: A European perspective. Migration and Health seminars 2021.
30. Esaiasson P, Johansson B, Ghersetti M, Sohlberg J. Kriskommunikation och segregation i en pandemi. Hur boende i utsatta områden informerade sig om coronaviruset våren 2020. (Translated: Crisis communication and segregation in a pandemic. How residents in vulnerable areas informed themselves about the coronavirus in spring 2020.) Report from The Department of Journalism, Media and Communication, University of Gothenburg; 2020.
31. Vingård E, Johansson B. Migration, arbetsmiljö och hälsa - En kunskapsöversikt. (Translation: Migration, work environment and health – a literature overview). Rapport 2012:4. The Swedish Work Environment Authority. Uppsala: Uppsala Universitet; 2012.
32. Zhang H, Nardon L, Sears G. Migrant workers in precarious employment. Journal of Equality, Diversity and Inclusion: An International Journal. 2021.
33. Anderson B. Migration, immigration controls and the fashioning of precarious workers. Work, employment society. 2010;24(2):300-17.
34. Abbasian S, Hellgren C. Working conditions for female and immigrant cleaners in Stockholm County: An intersectional approach. Nordic journal of working life studies. 2012;2(3):161-81.
35. Sanchez-Ramirez DC, Normand K, Zhaoyun Y, Torres-Castro R. Long-term impact of COVID-19: a systematic review of the literature and meta-analysis. Biomedicines. 2021;9(8):900.
36. O'Neill R. WHO knew. How the world health organization (WHO) became a dangerous interloper on workplace health and safety and COVID-19. NEW SOLUTIONS: A Journal of Environmental and Occupational Health Policy. 2020;30(3):237-48.
37. Birmingham Local Outbreak Engagement Board Covid-19 Overview. Data from 01/06/2020 – 18/01/2021. Birmingham; 2021.
38. Brekke J-P. Informing hard-to-reach immigrant groups about COVID-19—Reaching the Somali population in Oslo. Journal of Refugee Studies. 2022;35(1):641-61.

For peer review only

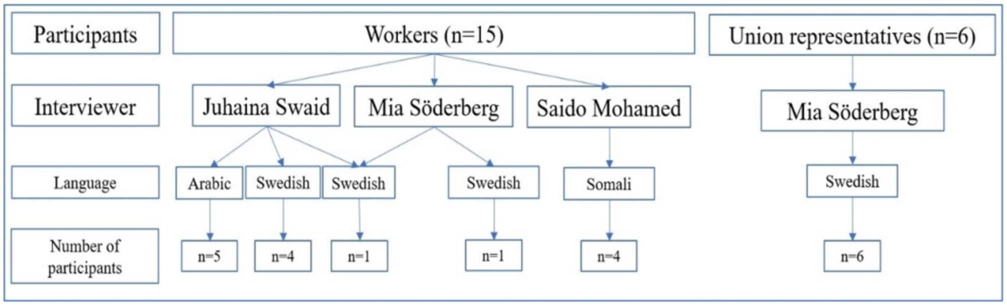

Figure 1. Languages used during interviews

162x48mm (220 x 220 DPI)

Appendix I

Descriptives of the study participants

Occupations of the workers
Tram or bus drivers (n=3)
Taxi drivers (n=3)
Property caretakers (n=2)
Cleaners (n=3)
Pizza bakers (n=4)
**Union representatives and their main occupation**
Transport union: Bus drivers (n=1)
Transport union: Taxi drivers (n=1)
Property service union: Property caretaker (n=1)
Property service union: General cleaners (n=3)
**Country of origin – workers**
Turkey (n=2)
Syria (n=4)
Iraq (n=2)
Palestine (n=2)
Somalia (n=5)
**Country of origin – Union representatives**
Sweden (n=3)
Norway (n=1)
Iran (n=1)
Serbia (n=1)

For peer review only

Reporting Checklist

Item 1. Title: Concise description of the nature and topic of the study. Identifying the study as qualitative or indicating the approach (e.g., ethnography, grounded theory) or data collection methods (e.g., interview, focus group) is recommended.
Response: page 1

Item 2. Abstract: Summary of key elements of the study using the abstract format of the intended publication; typically includes background, purpose, methods, results, and conclusions.
Response: page 2

Item 3. Problem Formulation: Description and significance of the problem/phenomenon studied; review of relevant theory and empirical work; problem statement.
Response: page 3-4

Item 4. Purpose or research question: Purpose of the study and specific objectives or questions.
Response: page 4

Item 5. Qualitative approach and research paradigm: Qualitative approach (e.g., ethnography, grounded theory, case study, phenomenology, narrative research) and guiding theory if appropriate; identifying the research paradigm (e.g., post-positivist, constructivist/interpretivist) is also recommended; rationale.
Response: page 6

Item 6. Researcher characteristics and reflexivity: Researchers' characteristics that may influence the research, including personal attributes, qualifications/experience, relationship with participants, assumptions, and/or presuppositions; potential or actual interaction between researchers' characteristics and the research questions, approach, methods, results and/or transferability.
Response: page 6

Item 7. Context: Setting/site and salient contextual factors; rationale.
Response: There is no relevant guideline

Item 8. Sampling strategy: How and why research participants, documents, or events were selected; criteria for deciding when no further sampling was necessary (e.g., sampling saturation); rationale.
Response: page 4-5

Item 9. Ethical issues pertaining to human subjects: Documentation of approval by an appropriate ethics review board and participant consent, or explanation for lack thereof; other confidentiality and data security issues.
Response: page 17

Item 10. Data collection methods: Types of data collected; details of data collection procedures including (as appropriate) start and stop dates of data collection and analysis, iterative process, triangulation of sources/methods, and modification of procedures in response to evolving study findings; rationale.
Response: page 5

For peer review only

Item 11. Data collection instruments and technologies: Description of instruments (e.g., interview guides, questionnaires) and devices (e.g., audio recorders) used for data collection; if/how the instrument(s) changed over the course of the study.
Response: page 5

Item 12. Units of study: Number and relevant characteristics of participants, documents, or events included in the study; level of participation.
Response: Appendix I

Item 13. Data processing: Methods for processing data prior to and during analysis, including transcription, data entry, data management and security, verification of data integrity, data coding and anonymization / de-identification of excerpts.
Response: page 6

Item 14. Data analysis: Process by which inferences, themes, etc. were identified and developed, including the researchers involved in data analysis; usually references a specific paradigm or approach; rationale.
Response: page 6

Item 15. Techniques to enhance trustworthiness: Techniques to enhance trustworthiness and credibility of data analysis,(e.g., member checking, triangulation, audit trail); rationale.
Response: Page 6

Item 16. Synthesis and interpretation: Main findings (e.g., interpretations, inferences, and themes); might include development of a theory or model, or integration with prior research or theory.
Response: Page 7

Item 17. Links to empirical data: Evidence (e.g., quotes, field notes, text excerpts, photographs) to substantiate analytic findings.
Response: There is no relevant guideline

Item 18. Integration with prior work, implications, transferability, and contribution(s) to the field: Short summary of main findings, explanation of how findings and conclusions connect to, support, elaborate on, or challenge conclusions of earlier scholarship; discussion of scope of application/generalizability; identification of unique contribution(s) to scholarship in a discipline or field.
Response: Page 14

Item 19. Limitations: Trustworthiness and limitations of findings
Response: Page 16

Item 20. Conflicts of interest: Potential sources of influence or perceived influence on study conduct and conclusions; how these were managed.
Response: Page 17

Item 21. Funding: Sources of funding and other support; role of funders in data collection, interpretation, and reporting.
Response: Page 17

For peer review only
